# Global Regulatory Frameworks for Fermented Foods: A Review

**DOI:** 10.3389/fnut.2022.902642

**Published:** 2022-05-23

**Authors:** Arghya Mukherjee, Beatriz Gómez-Sala, Eibhlís M. O'Connor, John G. Kenny, Paul D. Cotter

**Affiliations:** ^1^Department of Food Biosciences, Teagasc Food Research Centre, Fermoy, Ireland; ^2^APC Microbiome Ireland, University College Cork, Cork, Ireland; ^3^Department of Biological Sciences, University of Limerick, Limerick, Ireland; ^4^VistaMilk SFI Research Centre, Cork, Ireland

**Keywords:** fermented foods, legislation, kombucha, regulation, fermented milk, functional foods, yogurt, Codex Alimentarius

## Abstract

In recent years, there has been a global resurgence of public interest in fermented foods. In parallel, there have been several new studies that associate the consumption of fermented foods with a variety of beneficial impacts. These combined developments have led to a renewed focus in research and innovation vis-à-vis fermented foods, particularly traditional fermented foods, with an aim to harness this information to develop novel fermented foodstuffs and ingredients and make them available in the market. Consequently, an ever greater and more diverse array of fermented foods, including functional fermented foods with health benefits, are becoming available for public consumption in global markets, with the number expected to grow substantially in the coming decade. This rapidly expanding portfolio of commercially available fermented foods has in turn required an evolution in the corresponding global regulatory frameworks. Due to the innovative and emerging nature of these foods, combined with historical differences in regulator approaches, significant disharmony exists across these frameworks, with individual nations and organizations often adopting unique approaches relating to the establishment of standards and specifications. In this review, we provide an overview of the current regulatory frameworks for a diversity of fermented foods across multiple jurisdictions, with special emphasis on differences in legislative structures and approaches, regulatory harmonization, and current legislative limitations. Overall, the review provides important perspective and context in relation to current global fermented food regulatory practices with possible directions and recommendations for future legislative efforts.

## Introduction

Fermented foods, defined as “foods made through desired microbial growth and enzymatic conversions of food components” (1), provide an important means via which humans can be exposed to live, and potentially beneficial, microbes. The emergence of fermented foods (FFs) containing diverse microbial populations as staples in human societies can be traced back to the transition from hunter-gatherer populations to more settled agriculture-based communities 15,000–20,000 years ago (2). FFs continue to be consumed globally, with some estimates suggesting that these foods constitute up to one third of the current human dietary intake (3–8). These foods are highly diverse and reflect differences in food substrate, cultural influences, geographical availability, as well as microbial heterogeneity (4–6). Microorganisms in fermented foods and beverages can be derived from either the autochthonous, indigenous microbiota of raw animal and plant substrates or starter culture(s) containing specific, functional microbes (9, 10). Historically, the development of FFs and fermentation processes were primarily aimed at improving food safety and shelf-life. However, such processes often contribute to favorable biochemical transformations of the substrate, thereby enriching their nutritional value, making them organoleptically acceptable while also providing microbes and metabolites that can contribute to health (1, 11, 12).

In recent years, there has been a global resurgence in the interest in fermented foods, especially in Western society where consumption levels had decreased in previous decades (2, 8). For instance, it was noted recently that the fermented beverage market had grown considerably in Australia, with the kombucha market seeing the largest growth (~174% growth between 2016 and 2019) (13). This is greatly influenced by the purported nutritional and health benefits offered by FFs, inferred through several epidemiological studies, randomized controlled trials, clinical reports, and animal and *in vitro* studies (1, 6, 7, 14–17). Indeed, evidence-based investigations have associated FFs with health promoting properties such as a reduction in the risk of cardiovascular disease (CVD) and certain cancers (18, 19), modulation of inflammation (18, 19), protection against infection (14, 19) and promotion of a healthy brain and gut (14, 18). The renewed focus on FFs can also be attributed to a global need for food conservation and sustainability, in that fermentative processes can be implemented to preserve food and minimize food waste. Beyond this, certain consumers may simply wish to experience new sensory avenues and hence may be interested in less mainstream FFs.

Ongoing efforts to characterize FF microbiomes, and their impact on host biomarkers as well as the gut microbiome, have allowed us to gain a better understanding of the contribution/potential contribution of fermented food microbes to health (1, 20–23). Notably, there is renewed focus in researching traditional fermented foods from different parts of the globe, particularly for possible novel beneficial microbes and/or health benefits (20, 24–27). Together, these developments have led to the emergence of several new varieties of FFs in the market, a number of which have associated health claims. FFs with health claims are termed functional fermented foods, which is in agreement with the definition of functional foods that states that “foods can be regarded as functional if they can be satisfactorily demonstrated to affect beneficially one or more target function in the body, beyond adequate nutritional effects, in a way relevant to an improved state of health and well-being and/or reduction of risk of disease” (28). The drive to develop and commercialize fermented products has, in turn, brought forth the need for new regulations on different fermented products and highlighted the need for increased incorporation of scientific findings into regulatory legislations and claims. This has also resulted in a renewed focus on regulations pertaining to safety, transparency and quality. These FF regulatory frameworks are frequently influenced by geographical, political and/or cultural priorities and have varying inspirations, provisions, and degrees of implementation. Indeed, such regulations often reflect an integrated scientific and political engagement involving diverse stakeholders to assimilate evidence from foods, dietary patterns and health into specific, culturally adjusted and actionable public health policies. Unfortunately, however, many global FFs regulations lack cohesion and harmony, and focus primarily on food safety related aspects (29).

**Figure 1:**
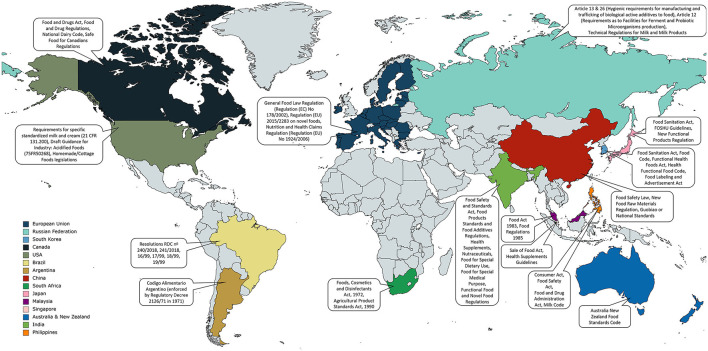
Global regulations for fermented foods. A selection of important regulations in relation to fermented foods is presented here. Countries are color-coded with pertinent regulations mentioned in linked callouts. This is not an exhaustive list of relevant regulations for fermented foods for countries shown in the figure. For more information of such regulations refer to the text and Supplementary Table 1. The figure was made using the open source, free-to-use online tool MapChart available at www.mapchart.net.

In this review, we present a comprehensive account of the current global regulatory context vis-à-vis FFs, with emphasis on functional FFs wherever applicable. In certain cases, regulations for probiotics, which are defined as “live microorganisms which when administered in adequate amounts confer a health benefit on the host” (30), have been discussed in relation to FFs with health claims. We discuss the various legislative approaches adopted by different countries across the globe in FF regulation along with relevant case studies, highlighting differences in approaches, socio-economic impact and outlook of such legislations (Figure 1, Supplementary Table 1). The descriptions of legislative approaches were deliberately categorized by nations/organizations to underscore the diversity in regulatory approaches. We have started by reviewing regulations from international organizations/unions such as the FAO and EU, followed by several countries in no particular order. Although regulatory frameworks relating to FFs have been previously reviewed for select countries (31–33), to our knowledge, this is the first comprehensive review of global legislative frameworks for FFs. It must be noted that we have concentrated on representative regulations from each country and the review is not an exhaustive source of FF regulations for said nations. Furthermore, unless otherwise specified, this review will focus only on regulations for products of non-alcoholic fermentation.

## Fermented Foods and the Codex Alimentarius

**Figure 2:**
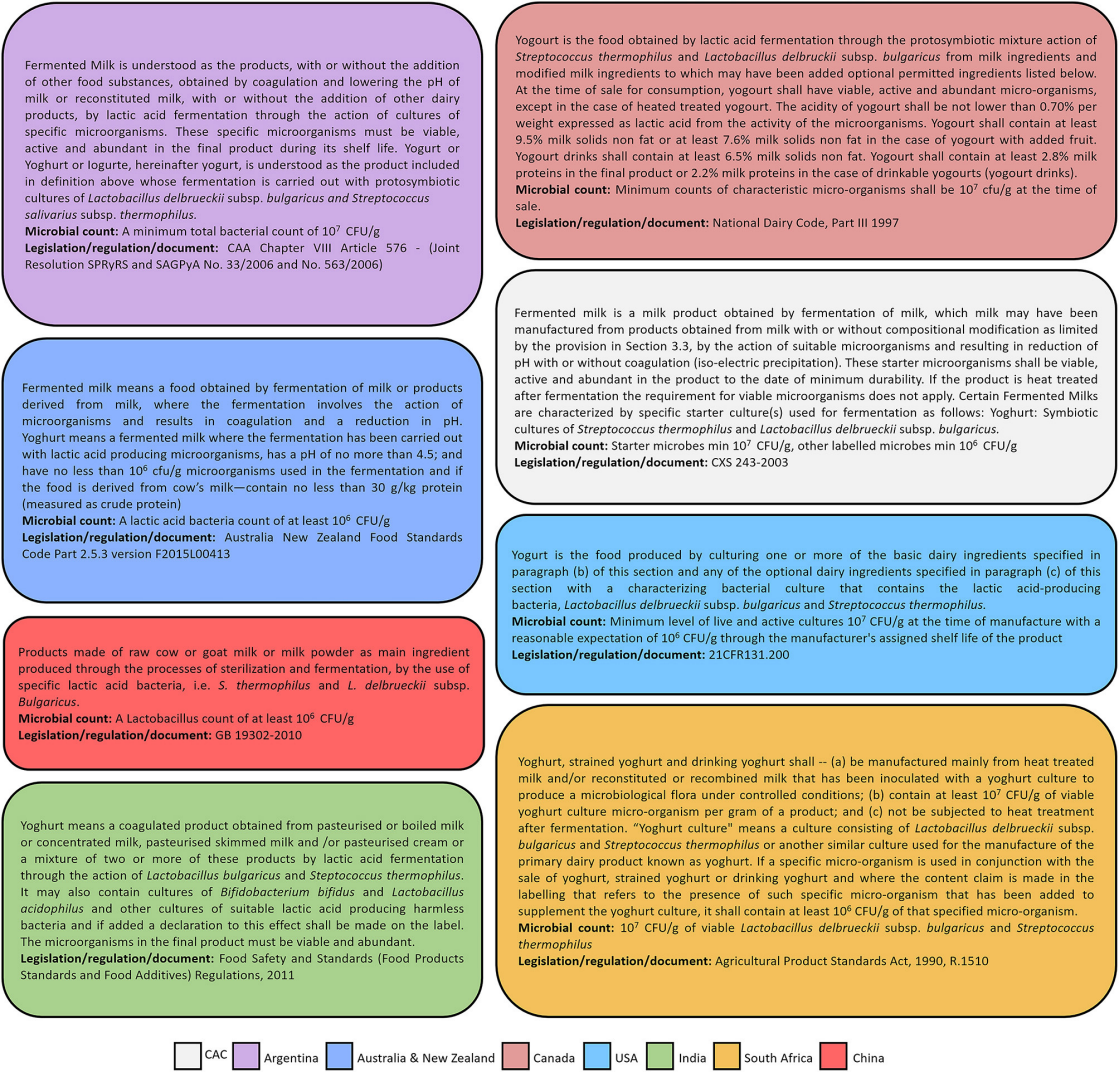
Global definitions of yogurt. Yogurt Standards for select countries and the Codex Alimentarius Commission (CAC) are described. Microbiological details including recommended species composition and microbial amounts are shown. CFU, colony forming units.

The Codex Alimentarius, or “Food Code,” is a collection of Standards, guidelines and codes of practice adopted by the Codex Alimentarius Commission (CAC), a fundamental part of the Food Standards Programme for the United Nations Food and Agriculture Organization (FAO) and the World Health Organization (WHO) (34). Most global legislative frameworks regarding foods are developed in broad agreement with recommendations of the CAC, thereby facilitating harmonization across governments/nations. However, FFs are not extensively represented in the Codex Alimentarius. The fourth revision of the Codex Alimentarius Standards for fermented milks (CXS 243-2003) was published in 2018 and provides standardized guidelines for yogurt, and a handful of other fermented or cultured milk products such as acidophilus milk, kefir and kumys (or koumiss) (Figure 2, Table 1). Fermented milk is described in CXS 243-2003 as “….a milk product obtained by fermentation of milk, which milk may have been manufactured from products obtained from milk with or without compositional modification…., by the action of suitable microorganisms and resulting in reduction of pH with or without coagulation. These starter microorganisms shall be viable, active and abundant in the product to the date of minimum durability. If the product is heat treated after fermentation the requirement for viable microorganisms does not apply” (Figure 2, Table 1). Importantly, the Standard describes the starter cultures for yogurt, alternate culture yogurt, acidophilus milk, kefir and kumys, among others (Table 1) (35). Additionally, the Codex Standard outlines the requirement for viable microbes (minimum 10^7^ colony forming units (CFU)/g of starter culture and 10^6^ CFU/g of microbes mentioned in labels) for sterilized and non-sterilized fermented milks. Notably, the Codex Standard for fermented milk Figure 2 products has facilitated harmonization of regulation vis-à-vis fermented dairy. For instance, similar definitions for describing dairy fermented foods, particularly yogurt are followed by several countries in corresponding regulations (Figure 2). Furthermore, most countries recommend a minimum of 10^7^ CFU/g starter culture bacteria in yogurt and a minimum of 10^6^ CFU/g of other microbes, if present, in line with the Codex Alimentarius Standards for fermented milk products (CXS 243-2003) (Figure 2) (32, 35). CXS 243-2003 additionally describes concentrated, flavored fermented milks and drinks based on fermented milks along with further elaboration on essential composition and quality factors, additives, contaminants, analytical methods and hygiene and labeling recommendations (35). Cheeses are separately covered under other Codex Standards (Supplementary Table 2).

**Table 1:** Codex Alimentarius Standards for fermented foods and beverages.

Fermented food/beverage (Codex Standard)^*^	**Standard type and published by**	**Definitions of fermented foods as per Codex Standards**	**References**
Fermented milks (CXS 243-2003)	Global Standard prepared by the Codex Committee on Milk and Milk Products (CCMMP)	*Fermented Milk* is a milk product obtained by fermentation of milk, which milk may have been manufactured from products obtained from milk with or without compositional modification, by the action of suitable microorganisms and resulting in reduction of pH with or without coagulation (iso-electric precipitation). These starter microorganisms shall be viable, active and abundant in the product to the date of minimum durability. If the product is heat treated after fermentation the requirement for viable microorganisms does not apply. Certain fermented milks are characterized by specific starter culture(s) used for fermentation as follows: Yogurt: Symbiotic cultures of *Streptococcus thermophilus* and *Lactobacillus delbrueckii* subsp. *bulgaricus*. Alternate culture yogurt: Cultures of *Streptococcus thermophilus* and any *Lactobacillus* species. Acidophilus milk: *Lactobacillus acidophilus*. Kefir: Starter culture prepared from kefir grains, *Lactobacillus kefiri*, and species of the genera *Leuconostoc, Lactococcus* and *Acetobacter* growing in a strong specific relationship. Kefir grains constitute both lactose fermenting yeasts *(Kluyveromyces marxianu*s) and non-lactose-fermenting yeasts *(Saccharomyces unisporus, Saccharomyces cerevisiae* and *Saccharomyces exiguu*s). Kumys: *Lactobacillus delbrueckii* subsp. *bulgaricus* and *Kluyveromyces marxianus*.	(35)
Kimchi (CXS 223-2001)	Global Standard prepared by the Codex Committee on Processed Fruits and Vegetables (CCPFV)	Kimchi is the product: (a) prepared from varieties of Chinese cabbage, *Brassica pekinensis* Rupr.; such Chinese cabbages shall be free from significant defects, and trimmed to remove inedible parts, salted, washed with fresh water, and drained to remove excess water; they may or may not be cut into suitable sized pieces/parts; (b) processed with seasoning mixture mainly consisting of red pepper (Capsicum annuum L.) powder, garlic, ginger, edible Allium varieties other than garlic, and radish. These ingredients may be chopped, sliced and broken into pieces; and (c) fermented before or after being packaged into appropriate containers to ensure the proper ripening and preservation of the product by lactic acid production at low temperatures.	(36)
Fermented cooked Cassava-based products (CODEX STAN 334R-2020)	Regional Standard published by the FAO/WHO Coordinating Committee for Africa (CCAFRICA)	Fermented cooked cassava-based products are presented in the form of cassava ball or sticks. These products are obtained from fresh cassava roots, peeled, cut, soaked in water for fermentation and pressed and dried before packaging and cooking. *Handling*: When cooking is done at the place of consumption, the uncooked product should be stored and transported under, time/temperature conditions that will not compromise the safety of the product.	(37)
Gochujang (CXS 294R-2009)	Regional Standard published by the FAO/WHO Coordinating Committee for Asia (CCASIA)	*Gochujang* is a red or dark red pasty fermented food manufactured through the following process: (a) Saccharified material is manufactured by saccharifying grain starch with powdered malt, or by cultivating *Aspergillus* sp. (which are not pathogenic and do not produce toxin) in grains; (b) Salt is mixed with the saccharified material obtained in the above (a). Subsequently, the mixture is fermented and aged; (c) Red pepper powder is mixed and other ingredients may be mixed with the mixture before or after the fermentation process (b) above; and (d) Processed by heat or other appropriate means, before or after being hermetically sealed in a container, so as to prevent spoilage.	(38)
Doogh (CXS 332R-2018)	Regional Standard published by the FAO/WHO CCASIA	Doogh is a “drink based on fermented milk” as defined in Section 2.4 of the *Standard for Fermented Milks*, obtained by mixing yogurt, as defined in Sections 2.1 and 3.3 of the same Standard, with potable water and optionally food grade salt or by mixing milk with potable water and sodium chloride prior to heat treatment and fermentation to give an end product with similar physical, chemical and organoleptic characteristics as the product defined under the provisions of this Standard. When doogh is produced by mixing milk with potable water, edible salt may be added before or after fermentation. The milk used for production of doogh may have been manufactured from products obtained from milk as specified in Section 2.1 of the *Standard for Fermented Milks*, with or without the compositional modification as limited by the provision in Section 3.3 in this standard. In the production of doogh, non-dairy ingredients, other than potable water, as well as various dairy ingredients/dairy products are used according to Sections 3 and 4.	(39)
		The typical starter microorganisms used in production of doogh are traditional yogurt bacteria: *Streptococcus thermophilus* and *Lactobacillus delbrueckii* subsp. *bulgaricus*. Microorganisms other than those constituting the specific starter cultures may be added. If the product is heat treated after fermentation, the requirement for viable microorganisms does not apply. Heat treatment after fermentation does not apply for “probiotic” doogh (doogh containing probiotic microorganisms). Doogh without added flavorings/flavor is called “plain doogh.” Doogh with flavors in the form of essences or extracts (such as menthol, ziziphora or wild thyme, pennyroyal and cucumber) or with different natural flavorings such as aromatic herbs, spices and condiments is known as “flavored doogh.” “Carbonated/Uncarbonated” and “Heat treated/Un-heat treated” dooghs represent those that contain/do not contain carbon dioxide and those with heat treatment/without heat treatment after fermentation, respectively. Doogh may be produced and displayed as powder (dried doogh) for special applications and demands.	
Fermented Soybean Paste (CXS 298R-2009)	Regional Standard published by the FAO/WHO CCASIA	Fermented Soybean Paste is a fermented food whose essential ingredient is soybean. The product is a paste type which has various physical properties such as semi-solid and partly retained shape of soybean and which is manufactured from ingredients such as soybeans, salt, potable water, naturally occurring or cultivated microorganisms (*Bacillus* spp. and/or *Aspergillus* spp., which are not pathogenic and do not produce toxins), grains and/or flour (wheat, rice, barley, etc.), yeast and/or yeast extracts, *Lactobacillus* and/or *Lactococcus, d*istilled ethyl alcohol derived from agricultural products (tapioca, sugar cane, sweet potato, etc.), sugars, starch syrup or natural flavoring raw materials (powder or extract from dried fish or seaweed, spices and herbs, etc.) through the following processes: (a) boiled or steamed soybeans, or the mixture of boiled or steamed soybeans and grains, are fermented with naturally occurring or cultivated microorganisms; (b) mixed with salt or brine and others; (c) the mixture or solid part of the mixture shall be aged for a certain period of time until the quality of the product meets hygienic requirements; and d) processed by heat or other appropriate means, before or after being hermetically sealed in a container, so as to prevent spoilage.	(40)
Tempe (CXS 313R-2013)	Regional Standard published by the FAO/WHO CCASIA	Tempe is a compact, white, cake-form product, prepared from dehulled boiled soybeans through solid state fermentation with *Rhizopus* spp. Essential ingredients include: (a) Soybeans (any variety); (b) Mold of *Rhizopus* spp. (*R. oligosporus,R. oryzae* and/or *R. stolonifer*) mix with cooked rice powder, rice bran powder and/or wheat bran powder as an inocula. Color: White color of luxurious growth of mycellium of Rhizopus spp. Flavor: Characteristic of tempe flavor, nutty, meaty, and mushroom-like. Odor: Characteristic of fresh tempe odor without ammonia smell.	(41)

* *CODEX STAN and CXS are equivalent designations*.

Codex Alimentarius Standards for kimchi (CXS 223-2001) have been recently revised, having first been published in 2001 (Table 1) (36). The Codex Standard defines kimchi in terms of its three-step preparation with fermentation, ripening and preservation constituting the final step (Table 1). It additionally specifies Chinese cabbage, seasoning mixtures and salt as essential ingredients, with “red color originating from red pepper,” a hot, salty and/or sour taste, and “a reasonably firm, crisp and chewy” texture specified as important organoleptic criteria. However, the text does not cover details relating to the microorganisms that should be present, other than a brief focus on microbiological safety and hygiene (36). Similar to the Codex Standard for fermented milks, CXS 223-2001 additionally describes the ingredients that are permitted, quality criteria, analytical methods, weights/measures, contaminants and recommendations for hygiene and labeling.

Besides Codex Standards for fermented foods approved by the CAC, several Regional Standards have been developed by the FAO/WHO Regional Coordinating Committees. Most of these Regional Standards relate to traditional FFs and beverages from their corresponding regions, which are consumed to only a limited degree in other parts of the world. Currently available Regional Codex Standards for FFs include fermented cooked cassava-based products (CXS 334R-2020) from Africa, and doogh (CXS 332R-2018), fermented soybean (CXS 298R-2009), gochujang (CXS 294R-2009), and tempe (CXS 313R-2013) from Asia (Table 1). Doogh is described as a “drink based on fermented milk” with *Streptococcus thermophilus* and *Lactobacillus delbrueckii* subsp. *bulgaricus* fermented yogurt listed as raw material; the definition also allows for use of other starter cultures, addition of flavor compounds, vegetables and dietary fibers (Table 1) (39). The Codex Standard for gochujang, a dark red pasty FF popular in Korea, describes it as a product of fermentation of saccharified material with non-harmful, non-toxin producing strains of *Aspergillus* sp. (Table 1) (38). Optional ingredients for gochujang in the Standard include powdered meju, a microbially fermented soybean product along with fermented soybean paste, fermented wheat protein and fermented rice, among others. The Codex Standard for fermented soybean paste (CXS 298R-2009), last amended in 2020, defines the food as fermented by either autochthonous, naturally occurring or cultured microbes (non-pathogenic, non-toxic strains of *Bacillus* sp. and/or *Aspergillus* sp.) with soybean being an essential component (Table 1) (40). Yeasts, yeast extracts, *Lactobacillus* and/or *Lactococcus* strains are mentioned as optional ingredients along with seaweed, spices, and herbs, among others. Tempe is defined in the Codex Standards as “a compact, white, cake-form product, prepared from dehulled boiled soybeans through solid state fermentation with *Rhizopus* spp.” (CXS 313R-2013). It further specifies the essential composition of tempe as being comprised of soybeans and a “mold of *Rhizopus* spp. (*R. oligosporus, R. oryzae*, and/or *R. stolonifer*) mix with cooked rice powder, rice bran powder and/or wheat bran powder as an inocula” (Table 1) (41). No additives are allowed in tempe as per CXS 313R-2013, with the texture defined as being “compact and not easily disintegrated upon cutting with knife.” CXS 334R-2020, the Codex Standard for fermented cooked cassava-based products defines the food as being obtained from fresh cassava roots (*Manihot esculenta* Crantz) with subsequent fermentation in water, pressing, drying and cooking; no microbiological information is available (Table 1) (37). For each of the fermented foods, the regional Standards provide additional information relating to standards for additives including flavor enhancers and preservatives, contaminants, weights and measures, analytical methods and recommendations for hygiene and labeling. As of 14th February 2022 and based on the information available on the FAO website, no fermented food related Regional Codex Standards have been published by the Coordinating Committees of Europe, North America and South America. A Codex Standard for kombucha does not currently exist. However, a Standards and Specification document for kombucha prepared by Uganda has been discussed at the World Trade Organization (WTO). The draft Standard specifies quality requirements for chemical (heavy metals, pesticides) and microbiological contaminants, analytical parameters (alcohol content, acidity from acetic acid, acidity in lactic acid and total sugar) and analytical methods, along with other details (33).

## Fermented Food Regulatory Frameworks in Europe

### The European Union

In Europe, fermented foods, including cultured milks, fall within the scope of the General Food Law Regulation (Regulation (EC) No 178/2002; current consolidated version 26 July, 2019), which is designed “to ensure a high level of protection of human life and consumers interests in relation to food, while ensuring the effective functioning of the internal market” (Figure 1, Supplementary Table 1) (42). Microorganisms that are used in food production including fermented foods, also known as food cultures, are considered as a category of food ingredients in the EU—one with a long history of use in diverse food products. However, despite their importance, food cultures are not defined in EU legislation. Similar to other food ingredients, food cultures are subject to fulfilling requirements set out in the General Food Law, Article 14 which states: “Food shall not be placed on the market if it is unsafe and it is the food business operator's responsibility for ensuring food safety” (42). Food cultures used in fermentation, however, are not subject to premarketing regulations in the EU, unless it is regarded as novel in the European single market and to its consumers.

Novel Foods are covered by Regulation (EU) 2015/2283 (implemented on 25 November 2015, current consolidated version 27/03/2021), in amendment of Regulation (EU) 1169/2011 and repealing Regulation (EC) 258/97 of the European Parliament and the Council and Commission Regulation (EC) 1852/2001 (43). Under the regulation, any food without a “significant” history of consumption in the EU before May 15, 1997 may be considered a novel food. This category covers new foods, food from new sources, new substances used in food as well as new ways and technologies for producing food. The law is therefore highly relevant for new enterprises and start-ups, especially those involved in the development and production of innovative functional foods or marketing traditional FFs from non-EU countries. It is important to note that starter cultures in fermented foods also need to fulfill the requirements of the Novel Foods Regulation because food consisting of, isolated from or produced from microbes is considered a novel food if not used within the Union prior to 1997 [Article 3, 2, (a), (ii) of Regulation 2015/2283]. Applications made under the regulation will be processed by the European Commission, thereby harmonizing and centralizing the novel foods regulations that were previously managed by Member States (under Regulation (EC) 258/97) and will significantly reduce time taken to acquire an approval for novel foods. Before it can be placed on the market, the pre-market authorization procedure for novel foods includes a thorough risk assessment by the European Food Safety Authority (EFSA) and production of a scientific opinion as per Article 29 (1) (a) of Regulation (EC) No 178/2002 (42). EFSA has published guidance documents on novel and traditional foods in relation to risk assessment pursuant to Regulation (EU) 2015/2283. If found to meet the criteria set forth in regulations 2015/2283, the product may be authorized for placement in the EU market. Since 1997, no new microorganisms used as live food cultures have been evaluated and authorized under the novel food directive in the EU. Genetically modified microbes used in food cultures are also considered novel food components, but in this case the novel food has to demonstrate compliance with regulations on genetically modified organisms (Directive 2001/18/EC, Regulation (EC) No 1829/2003, and Commission Regulation (EC) No 641/2004). For traditional foods originating in non-EU countries, which are often FFs and will be considered as novel foods, Article 14 for Regulation (EU) 2015/2283 for novel foods provides an alternative route for authorization and subsequent access to the European single market. Among others, evidence for the safe consumption of the traditional food as a customary diet by a significant number of people in at least one country outside of the EU for a period of at least 25 years must be presented [rules for implementation detailed in Regulation (EU) 2017/2468]. The procedure for novel foods authorization under Regulation (EC) No 258/97 by the EU has been described previously by Bell *et al* in relation to fermented soybean extracts (31). A list of novel foods involving microbes/microbial products approved by the EU over the years is presented in Table 2.

**Table 2:** Novel foods involving microbes/fermented products approved in the EU under the EU regulation for novel foods.

**Authorized novel food**	**Description/Designation**	**Implementation Decisions**	**Live microbes**
UV-treated baker's yeast (*Saccharomyces cerevisiae*)	*Saccharomyces cerevisiae* or baker's yeast is treated with ultraviolet light to induce the conversion of ergosterol to vitamin D2 (ergocalciferol). The yeast concentrate is blended with regular baker's yeast in order not to exceed the maximum level in the pre-packed fresh or dry yeast for home baking. The designation of the novel food on the labeling of the foodstuffs containing it shall be “Vitamin D yeast” or “Vitamin D2 yeast.”	Commission Implementing Regulation (EU) 2018/1018 of 18 July 2018 authorizing an extension of use of UV-treated baker's yeast (Saccharomyces cerevisiae) as a novel food under Regulation (EU) 2015/2283	Very low, within EFSA safety standards set for the novel food
Dried *Euglena gracilis*	The novel food is dried whole cell *Euglena*, which is the dried biomass of the microalga *Euglena gracilis*. The novel food is produced by fermentation followed by filtration and a heat-killing step of the microalga to ensure the absence of viable *Euglena gracilis* cells in the novel food. The designation of the novel food on the labeling of the foodstuffs containing it is “dried biomass of *Euglena gracilis* algae.”	Commission Implementing Regulation (EU) 2020/1820 of 2 December 2020 authorizing the placing on the market of dried *Euglena gracilis* as a novel food under Regulation (EU) 2015/2283	Very low, within EFSA safety standards set for the novel food
*Yarrowia lipolytica* yeast biomass	The novel food is the dried and heat-killed biomass of the yeast *Yarrowia lipolytica*. The designation of the novel food on the labeling of the foodstuffs containing it is “*Yarrowia lipolytica* yeast heat-killed biomass”'	Commission Implementing Regulation (EU) 2019/760 of 13 May 2019 authorizing the placing on the market of *Yarrowia lipolytica* yeast biomass as a novel food under Regulation (EU) 2015/2283	Very low, within EFSA safety standards set for the novel food
Fermented black bean extract	Fermented black bean extract (Touchi extract) in the form of a fine light-brown protein-rich powder obtained by water extraction of small soybeans (*Glycine max* (L.) Merr.) fermented with *Aspergillus oryzae*. The extract contains an α-glucosidase inhibitor. The designation of the novel food on the labeling of the foodstuffs containing it is ‘Fermented black bean (Soya) extract” or ‘Fermented Soya extract'	Commission Implementing Decision of 9 August 2011 authorizing the placing on the market of fermented black bean extract as a novel food ingredient under Regulation (EC) N° 258/97 of the European Parliament and of the Council	Very low, within EFSA safety standards set for the novel food
Heat-treated milk products fermented with *Bacteroides xylanisolvens*	Semi-skimmed milk (between 1.5 and 1.8% fat) or skimmed milk (0.5% fat or less) is pasteurized or ultra-heat-treated before starting the fermentation with *Bacteroides xylanisolvens* (DSM 23964). The resulting fermented milk product is homogenized and then heat-treated to inactivate *Bacteroides xylanisolvens* (DSM 23964). The final product does not contain viable cells of *Bacteroides xylanisolvens* (DSM 23964).	Commission Implementing Decision (EU) 2015/1291 of 23 July 2015 authorizing the placing on the market of heat-treated milk products fermented with Bacteroides xylanisolvens (DSM 23964) as a novel food under Regulation (EC) No 258/97	Very low, within EFSA safety standards set for the novel food
*Clostridium butyricum*	*Clostridium butyricum* (CBM-588; depository number FERM BP-2789) is a Gram-positive, spore-forming, obligate anaerobic, non-pathogenic, non-genetically modified bacterium. The designation of the novel food on the labeling of the foodstuffs containing it is “*Clostridium butyricum* MIYAIRI 588 (CBM 588)” or “*Clostridium butyricum* (CBM 588)”	2014/907/EU: Commission Implementing Decision of 11 December 2014 authorizing the placing on the market of *Clostridium butyricum* (CBM 588) as a novel food ingredient under Regulation (EC) No 258/97	Viable spores of *C. butyricum*
Fermented soybean extract	Fermented soybean extract in the form of an odorless milk-white colored powder. It is comprised of 30 % fermented soybean extract powder and 70 % resistant dextrin (as carrier) from corn-starch, which is added during the processing. Vitamin K2 is removed during the manufacturing process. Fermented soybean extract contains nattokinase isolated from natto, a foodstuff produced by the fermentation of non-genetically modified soybeans [*Glycine max* (L.)] with a selected strain of *Bacillus subtilis* var. natto. The designation of the novel food on the labeling of the foodstuffs containing it is “Fermented soybean extract.”	Commission Implementing Decision (EU) 2017/115 of 20 January 2017 authorizing the placing on the market of fermented soybean extract as a novel food ingredient under Regulation (EC) No 258/97	Very low, within EFSA safety standards set for the novel food
Dried *Tetraselmis chuii* microalgae	The dried product is obtained from the marine microalgae *Tetraselmis chuii*, cultivated in sterile sea water in closed photobioreactors insulated from the outside air. The designation of the novel food on the labeling of the foodstuffs containing it is “Dried microalgae *Tetraselmis chuii*” or “Dried microalgae *T. chuii*.”	Spanish Agency for Food Safety and Nutrition authorizes the product as novel food and compliant with Regulation (EC) No 258/97	Very low, within EFSA safety standards set for the novel food
Selenium-containing yeast (*Yarrowia lipolytica*) biomass	The novel food is the dried and heat-killed selenium-containing biomass of the yeast *Yarrowia lipolytica*. The novel food is produced through fermentation carried out *Yarrowia lipolytica* in the presence of sodium selenite followed by a number of purification steps including a heat-killing step of the yeast to ensure the absence of viable *Yarrowia lipolytica* cells in	Commission Implementing Regulation (EU) 2020/1993 of 4 December 2020 authorizing the placing on the market of selenium-containing yeast (*Yarrowia lipolytica*) biomass as	Very low, within EFSA safety standards set for the novel food
	the novel food. The designation of the novel food on the labeling of the foodstuffs containing it is “selenium-containing yeast (*Yarrowia lipolytica*) biomass.”	a novel food under Regulation (EU) 2015/2283	
Chromium-containing yeast (*Yarrowia lipolytica*) biomass	The novel food is the dried and heat-killed chromium-containing biomass of the yeast *Yarrowia lipolytica*. The novel food is produced through fermentation carried out *Yarrowia lipolytica* in the presence of chromium chloride followed by a number of purification steps and a heat-killing step of the yeast to ensure the absence of viable *Yarrowia lipolytica* cells in the novel food. The designation of the novel food on the labeling of the foodstuffs containing it is“chromium-containing yeast (*Yarrowia lipolytica*) biomass.”	Commission Implementing Regulation (EU) 2020/1822 of 2 December 2020 authorizing the placing on the market of chromium-containing yeast (*Yarrowia lipolytica*) biomass as a novel food under Regulation (EU) 2015/2283	Very low, within EFSA safety standards set for the novel food

*The list does not include specific microbial products produced using microbial fermentation and subsequently extracted (Regulation (EC) No. 258/97 and Regulation (EU) 2015/2283 of the European Parliament and of the Council on Novel Foods; full list available at: https://ec.europa.eu/food/food/novel-food/authorisations/union-list-novel-foods_en)*.

Communications regarding the nutritional and health effects of foods, including fermented foods, is regulated by the Nutrition and Health Claims Regulation 1924/2006 (NHCR) (Figure 1, Supplementary Table 1). The regulation requires food business operators to obtain prior authorization from the EU Commission in order to communicate the beneficial effects of their products through labels or advertising, i.e., obtaining a health claim approval. Thus, fermented foods with probiotics or claiming other health benefits, must apply for approval from EFSA before they can be marketed in the EU single market. Under the Food Law, manufacturers can optionally label their foods with constituent microbes when no corresponding health claim is made. Of more than 400 health claim applications for probiotics and fermented foods submitted to the European Commission, only one has been authorized. This involves an article 13(1) health claim under the NHCR that states that “live cultures in yogurt improve lactose digestion of the product in individuals who have difficulty digesting lactose” with said yogurt cultures being L. delbrueckii subsp. bulgaricus and S. thermophilus when present at a minimum concentration of 10^8^ CFU/g (44). All other claim applications, e.g., relating to improved gut or immune function, have been rejected, with the most frequently cited reason for rejection being insufficient demonstration of claimed health benefit.

Recent regulatory developments around probiotics in Europe may have important implications for FFs, particularly those with health claims. Currently, most EU countries consider the term “probiotic” a health claim and several do not even allow unspecified claims such as “contains live bacteria.” However, in the absence of a harmonized and well-defined policy, probiotics and functional FFs are subject to national provisions and several Member States are using the term “probiotics” in more generic terms. Recently, the Spanish Agency for Food Safety and Nutrition (AESAN) has expressed willingness to accept the use of the term “probiotic” on labels of food and food supplements produced and commercialized in the country without the authorization of any health claim until a uniform criterion for probiotics is generated by the Member States of the EU. AESAN has provided a 3-fold explanation for such a decision. Firstly, it recognizes that there are different interpretations for use of the term “probiotic” among the Member States leading to a non-harmonized European Union market. Secondly, it argues that implementation of provisions set for the use of the term “probiotic” in the NHCR Guidance is legally non-binding on EU Member States. Thirdly, it refers to the “principle of mutual recognition” established in the EU Treaty (Regulation EU 2019/515), whereby any product legally marketed and sold in one EU Member State may be sold in others as well. AESAN elaborates that due the non-harmonized European Market vis-à-vis probiotics, adjacent markets are already using the term “probiotic” in a more generic fashion and in turn marketing their products in Spain, to the detriment of Spanish industry and market. Previously, France, Portugal and Belgium have allowed the use of the term “probiotic” as a non-specific health claim, when accompanied by a specific health claim. More recently, Italy has indicated that the term “probiotic” might be used for food and food supplements of probiotic microorganisms traditionally used for intestinal microbiota balance. Additionally, the Czech Republic has issued national guidelines allowing the use of the term “contains probiotics” as a nutrition claim, subject to the fulfillment of the conditions of use for nutrition claims defined in the NHCR. The Netherlands, Denmark and Poland have recently stated that in the future they will consider “probiotics” as a mandatory category term for dietary supplements but not for other foods or food ingredients. These regulatory reforms, understandably, will have significant impact on the development, commercialization and sale of FFs with health claims in the EU. Indeed, probiotic regulations are often closely linked with regulations for FFs with health claims, as we will see below. While deviation from the definition of probiotics with regards to labeling and communication as set forth by the NHCR in national policies of the Member States would certainly re-open the market for more products, it essentially creates a fragmented EU marketplace for probiotic products including functional FFs. Notably, this can create considerable confusion in differentiating “definitive” and “generic” probiotics/functional FFs.

### The Russian Federation

Within the Russian Federation, relevant legislation relating to FFs include the “Requirements for Ferments and Enzyme Preparations” (Article 12) and “Requirements as to Facilities for Ferment and Probiotic Microorganisms production” (articles 13 and 26) detailed in the Federal Law “Hygienic requirements for manufacturing and trafficking of biological active additives to food” (SanPiN 2.3.2 1290-03) (45) (Figure 1, Supplementary Table 1). Additionally, the Federal Law on “Technical Regulations for Milk and Milk Products” (88-FZ; amended in 163-FZ on 22nd August 2010) outlines safety and compliance requirements including processes of manufacture, packing, marking, storage, transportation, sale and disposal of milk and dairy products in the Russian Federation (46). Among other things, the law defines various milk products, including fermented dairy products such as yogurt, ayran, kumiss, varenet, buttermilk, curd, ryazhenka, sour cream and diverse cheeses. It also provides physicochemical, organoleptic and microbiological parameters for the identification and assessment of most dairy products.

## Fermented Foods Regulatory Frameworks in North America

### United States of America

In US, all foods are subject to both Federal and corresponding State Laws. While the US Food and Drug Administration (FDA) has put regulations in place pertaining to acidified foods including yogurt, it does not regulate other FFs since they have not found any cases of untoward consequences from consumption of same (47). In the US, the FDA describes yogurt as a dairy product produced upon fermentation of milk by *L. bulgaricus* and *S. thermophilus*; the regulation allows addition of probiotic cultures and other lactic acid bacteria (21 CFR 131.200) (Figures 1, 2, Supplementary Table S1). In the same regulation, minimum requirements for cultured milks are set to: at least 3.25% milk fat, 8.25% milk solids-nonfat, and a titratable acidity of 0.5% expressed as lactic acid, with allowances for additional fortification with vitamins A and D (32). On September 2010, the US FDA published the “*Draft Guidance for Industry: Acidified Foods”* (75FR50268), which provided recommendations relating to the manufacturing, storage, packaging distribution, and quality control for acidified foods including eligible fermented foods. However, the guidance was later partially withdrawn on December 2015 (80FR81550) as several of the addressed topics are presently dealt through other documents. Acidified foods must also be compliant with other Federal Laws including Good Manufacturing Practice (21 CFR part 117 Subpart B), Acidified Foods Regulation (21 CFR 114), Emergency Permit Control (21 CFR 108), Thermally Processed Low-Acid Foods Packaged in Hermetically Sealed Containers Regulation (21 CFR 113), and FDA Acidified and low-acid canned foods guidance and regulations (FDA-2017-D-3716, FDA-2013-D-1622), among others. The regulation for yogurt, even though an acidified food, is provided through the “Requirements for Specific Standardized Milk and Cream” (21 CFR part 131.200); the regulation defines yogurt in terms of microbiological and nutritive parameters, and outlines labeling requirements, assessment methods, and acceptable additives, among others.

The Homemade Foods or Cottage Foods legislations in various US States are another set of important regulatory framework with respect to FFs (Figure 1, Supplementary Table 1). All states in the US except New Jersey have some form of Cottage Foods legislation. These laws regulate how home-based food businesses are operated in the US, allowing such businesses to sell products at farmers' markets and can even differ within different counties. However, most Homemade Food Laws limit the sale of potentially hazardous foods that include microorganisms and require controlled temperature and time to prepare [e.g., California Homemade Food Act (AB 1616), California Homemade Food Operations Act 2018 (AB 626), Texas Cottage Food Law 2011 (SB81), amended Texas Cottage Bill Law 2013 (HB970)]. Several states are in the process of expanding the range of cottage foods included in these bills, including foods produced through microbial action, to allow further expansion of the Cottage Food enterprises. For example, in 2019, Texas amended the Cottage Food Law (SB 572) to include more foods such as fermented vegetables with a pH of 4.6 or less, among others. FFs such as kombucha remain excluded to date. The Homemade Foods Bill in Texas (HB 1926) additionally outlines safety requirements for the Cottage Foods Laws including sanitation measures, health restrictions and record-keeping and labeling, among others. Separate from the Cottage Foods program, some states have introduced Food Freedom Laws that allow residents to sell almost any kind of homemade food with the exception of meats, including FFs such as kefir, kombucha and sauerkraut. Contrary to the Cottage Foods Laws, the Food Freedom Laws do not have a limitation on sale, licensing, permissions, and inspection requirements, and are therefore comparatively more lenient. One important consideration of these laws is that the product must be sold to “informed end consumers.” States that have Food Freedom Laws in place include Wyoming (48), North Dakota and Utah, while Alaska and Mississippi are considering similar laws. The growing consumer interest in locally made, organic foods including fermented foods has buoyed the American cottage foods sector, with the value of home food businesses growing from $5 billion in 2008 to nearly $20 billion in 2016 with, for example, Wyoming's farmer's markets growing by 70% since the introduction of the Food Freedom Act in 2015 (48). Further information on the recent legislative development around fermented foods in the US can be accessed through the Fermentation Association (https://fermentationassociation.org/head-food-and-beverage-laws-passed-in-2021/, https://fermentationassociation.org/food-and-beverage-laws-passed-in-2021-part-2/).

The development of US legislation involving “gluten-free” health claims provide an example of how legislative frameworks can be developed for specific health claims, where a certain demography may be at risk. In this case, the vulnerable demography refers to individuals suffering from celiac disease, which is a hereditary, chronic inflammatory disorder of the small intestine that is triggered by gluten intake. An associated final rule was published in the US Federal Register by the FDA in 2013, in which the term “gluten-free” for voluntary use in labeling foods was defined (21 CFR 101.91; document no. 78 FR 47154). The rule provides protection to individuals with celiac disease through enforcement of truthful and accurate labeling of relevant information. More recently, in 2020, the US FDA released a final rule to establish compliance requirements for fermented and hydrolyzed foods that bear the “gluten-free” claim. The final rule, titled “Gluten-Free Labeling of Fermented or Hydrolysed Foods,” covers fermented foods such as yogurt, sauerkraut, pickles, cheese, as well as green olives, FDA-regulated beers and wines, and hydrolysed plant proteins. Under this rule, FDA will determine compliance based on records kept by the manufacturer to show that their foods are gluten-free before fermentation or hydrolysis occurs (21 CFR 101; document no. 85 FR 49240).

In recent years, certain commercial groups have become involved in driving codes of practice aimed at the regulation of FFs. Kombucha Brewers International (KBI) is a US-based trade association with 40 initial founding companies and 300 brewery members. It represents global commercial interests with respect to kombucha and provides a kombucha code of practice to ensure food safety, high standards of quality and transparent communication to enable consumers to make an informed choice (33). The KBI code of practice defines ingredients, processing, and manufacturing steps for kombucha along with recommendations for hygiene and labeling (49). It additionally specifies the kombucha analytical profile through standardized chemical and microbiological levels in the fermented beverage. Importantly, KBI has a certification and seal program for commercially available kombucha that indicates compliance with high levels of manufacturing standards and food safety (33). In the past decade, KBI has had significant involvement in the development and implementation of laws concerning kombucha in the United States. In 2015, KBI initiated an Association of Official Analytical Chemists Working Group in discussion with the US Alcohol and Tobacco Tax and Trade Bureau to determine the best analytical approach for ethanol testing in kombucha. Additionally, it was involved in lobbying for the KOMBUCHA (*K*eeping *O*ur *M*anufacturers from *B*eing *U*nfairly taxed while *C*hampioning *H*ealth) Act that demanded an update to the Internal Revenue Code to exempt kombucha from federal excise taxes and regulations intended for beer (27 CFR Part 5). More specifically, an increase in the allowable alcohol by volume (ABV) for kombucha only from 0.5 to 1.25% was demanded. This culminated in the drafting of the kombucha law by US senators in association with KBI in 2017. In the proposed law, kombucha was defined as a beverage that “(a) is fermented only by a symbiotic culture of bacteria and yeast, (b) contains no more than 1.25% alcohol by volume, (c) is sold or offered for sale as kombucha, and (d) is derived from sugar, malt or malt substitute, tea or coffee and no more than 20% of other healthy ingredients.” However, the law has yet to be ratified by the US senate (50). A procedural guidance and risk analysis document for kombucha fermentation was also published by the National Environmental Health Association (NEHA), an US-based environmental services organization in 2013, under the Model Code of Food Administration and Medicines as stated by the FDA (51). The document elaborates Hazard Analysis & Critical Control Point (HACCP) based food safety management for the entire kombucha management chain and suggests pasteurization, refrigeration and addition of preservatives such as 0.1% sodium benzoate and 0.1% potassium sorbate as other methods to ensure beverage safety (52). The FDA Model Food Code recommends a pH of ≤4.2 as a critical limit, which, if not reached within 7 days of fermentation, may indicate contamination and qualifies the kombucha preparation for dismissal (52).

### Canada

In Canada, live bacterial cultures, including probiotics, are considered as food ingredients under the food provisions of the Food and Drug Regulation and can be added to foods (53). Foods containing microbes, such as FFs, are generally classified as foods and regulated similarly. Currently, the general provisions of the Food and Drugs Act and Regulations apply to both foods containing microbes and microbes represented as probiotics (53, 54). In cases where foods are represented as having therapeutic use or purpose, i.e., a health claim, the product is generally classified as a natural health product (NHP). Such NHPs can potentially be sold in food format, such as functional FFs, and are distinguished from general foods based on Health Canada's guidance document “Classification of Products at the Food-Natural Health Product Interface: Products in Food Formats.” The evaluation takes into consideration the nature of and risks associated with the microorganisms involved, the product's represented therapeutic use, history of use and public perception of the product's intended use, among others. All health claims made for foods, including probiotics and functional FFs, are subject to Subsection 5(1) of the Food and Drugs Act, which requires that all claims and representations on food products are backed by proper scientific evidence and will not create an erroneous impression about the product, thereby misleading the public (54). A guidance document posted by Health Canada titled “The Use of Probiotic Microorganisms in Food” explains the conditions under which health claims about probiotics, and as extension functional FFs, would be considered acceptable for food. This guidance document is used by the Canadian Food Inspection Agency (CFIA) to assess compliance of food products containing microbes represented as probiotics with the Food and Drugs Act and Regulations (55) (Figure 1, Supplementary Table 1). Notably, the Canadian government allows non-strain specific health claims for probiotics in foods when the product contains one or more authorized species (primarily *Lactobacillus* spp. and *Bifidobacterium* spp.) with a declared minimum viability level of 10^9^ CFU per stated serving size of food maintained throughout the product shelf-life (32, 56).

Canada provides federal regulations as Standards of Identity (SI; enforced by the CFIA) for a variety of foodstuffs including FFs, which have now been incorporated in the Safe Food for Canadians Regulations (SFCR) that came into force on January 15, 2019 (57). Labeling requirements for cheeses and certain prepackaged edible fermented meat products are covered in the SFCR, with references to the Standards of Identity where appropriate. For instance, fermented meats must declare “…is fermented and has a pH of 5.3 or less, and a water activity of 0.90 or less, at the end of the fermentation” on their products and abide by the set parameters (57). Curiously, Canada does not have federal regulations for fermented milk products beyond cheese (which are covered in Volume I of the Canadian SI), with standards and definitions for common fermented milks such as yogurt being absent in the SFCR as well as the SI. Instead, Part III of the National Dairy Code, a technical reference document, has been referred to as a guidance (and not regulation) for safe and suitable production of dairy products (58) (Figures 1, 2, Supplementary Table 1). The Code describes microbiological and nutritional compositions, labeling requirements and allowed additives for fermented dairy such as yogurt (or yogourt), buttermilk, sour cream and cultured cream and is largely aligned with the Codex Alimentarius standards for fermented milk products (CODEX STAN 243-2003) (Figures 1, 2) (35, 59). Importantly, there are no restrictions to the addition of other microorganisms beyond the yogurt starter cultures. It has been opined that such regulatory leniency is deliberate, as Canada reportedly came close to publishing federal standards for yogurt but did not follow through. Such a decision may have been taken in order to ensure that innovation, research and novel product development in the booming functional foods sector of Canada is not stifled, where functional yogurts represent an important fraction of commercially available functional foods. This allows manufacturers to market innovative products in Canada through creative communications of “implied” claims, thereby bypassing overly restrictive and often confusing regulatory frameworks for products with health claims, such as probiotics and NHPs. Canada's lenient approach to regulating FFs such as yogurt that have innovative potential is yet another example of how different countries have approached FF regulation divergently depending on current scientific understanding and economic interests, among other factors. Food safety guidance for kombucha preparation is available for commercial manufacturers in Canada through a document of recommendations from the Centre of Disease Control of British Columbia (BCCDC). These guidelines describe the possible biological and chemical risks and hazards in kombucha production, and emphasize alcohol content and pH as critical parameters that require close monitoring. According to BCDC recommendations, alcohol levels in kombucha should not exceed 1% and pH levels should not drop below 2.5 (60).

## Fermented Foods Regulatory Frameworks in AsiA

### Japan

In Japan, food safety and standards fall within the scope of the Food Sanitation Act (Act No. 233, 1947) (Figure 1, Supplementary Table 1). This includes composition and standards descriptions for food additives (“Specifications and Standards for Food and Food Additives, etc.”; Ministry of Health and Welfare Notification No. 370, 1959) and milk and milk products (Ministerial Ordinance on Milk and Milk products Concerning Compositional Standards, etc.; Ministry of Health and Welfare Ordinance No. 52, 1951). Although mostly lacking specific regulations for FFs, the latter does include certain requirements for cheeses and fermented milk products. All lactic acid bacteria are allowed in Japan as food cultures (FCs); they are regarded as foods and are listed in the “List of substance which are generally provided for eating or drinking as foods and which are used as a food additive” as “lactic acid bacteria concentrates” (61).

In 1991, Japan's Ministry of Health, Welfare and Labor formed the Food for Specified Health Uses (FOSHU) guidelines as a regulatory system for functional foods, including probiotic-containing FFs (62) (Figure 1, Supplementary Table 1). Foods found compliant with the FOSHU guidelines where active ingredients had been scientifically substantiated to provide health benefits, are approved to bear the FOSHU tag on the product label (63). Products do not require a clinical trial in Japan, unlike in the EU vis-à-vis EFSA, and government approval of the active ingredient is considered acceptable. Approximately 55% of health claims in FOSHU are related to improving GI tract health using probiotic lactobacilli and bifidobacteria, oligosaccharides and dietary fibers with most probiotic bacteria deployed in yogurt or yogurt-like fermented milks (62). Additionally, certain antihypertensive peptides originating from fermented milks and milk casein hydrolysates have also receive FOSHU approval. Registration of functional foods peaked in 2007, with a subsequent decline attributed to long approval periods, slow response to additional requests, a relatively narrow spectrum of acceptable health claims, and increasing financial infeasibility, among others. To counter this, the “Foods with Function Claims” or New Functional Products (NFP) Regulation was established in 2015, which incorporated a more flexible and broadened spectrum of acceptable health claims compared to FOSHU, did away with the requirement for government approval with companies required to be responsible for their products and accepted “scientific reviews” (systematic reviews) in lieu of clinical studies as proof of health claim. Relaxation in regulations resulted in the greater involvement of small- and medium-scale enterprises in the Japanese NFP market, which was estimated to be worth $1.8 billion in 2018, with the total functional foods market valued at $8 billion; as of 2019, 1,785 NFPs are available in Japan (62).

In order to boost the export and guarantee the safety of natto, one of Japan's iconic fermented foods, the Japanese Ministry of Agriculture, Forestry and Fisheries has started working with the Codex Alimentarius Commission (CAC) to develop the “Asian Regional Standards for Soybean Products Fermented using microorganisms Bacillus.” To this end, Japan will be working closely with other relevant stakeholders such as China, South Korea and Thailand. The Codex Standard will effectively involve most fermented soybean products similar to natto, such as South Korea's cheonggukjang, China's douchi, Thailand's thua nao sa and India/Nepal's kinema, among others. All of these foods involve fermentation using *Bacillus*, most commonly *Bacillus subtilis*. For now, the standard is meant to be regional, rather than international, as consumption of fermented soybean products is highest in the said regions. The draft standard is expected in 2022, while the finalized standard is expected to be formally adopted by 2024.

### China

The Food Safety Law is the overarching food regulatory legislation in China with detailed rules for food standards, food surveillance and assessment, and food import and export, among others (64). Currently, the law does not have any special provisions for FFs. The New Food Raw Materials Regulation (formerly “Novel Foods Regulation”) evaluates food ingredients not traditionally used in China and includes new microbes in foods (such as food cultures) and food processing and holds relevance with respect to FFs. From 2010 and as of May 2017, 35 microbial species had been approved for use in FF products (29). Additionally, three strains from Lallemand (*Lactobacillus helveticus* R0052, *Bifidobacterium infantis* R0033 and *B. bifidum* R0071), a private enterprise involved in developing specialty ingredients including probiotic and yeasts strains, were added to the novel food raw materials list in 2020. China also has “Guobiao” (GB) or ‘National Standards” for certain FFs; these Standards are equivalent to the ISO standards used in Western countries (Figure 1, Supplementary Table 1). The National Food Safety Standard - Fermented Milk [GB 19302-2010; corresponds to the Codex Standard for fermented milks (243-2003)] outlines the technical requirements including the acceptable organoleptic, contaminant, mycotoxin and nutritional metrics as well as microbial strains and loads for fermented milks and yogurt, including flavored alternatives (Figures 1, 2). No other variants of fermented dairy are discussed. Products named soy sauce are regarded as liquid seasonings obtained through the fermentation of soybean (or cereal grains) in China, and standards for the same are regulated through National Food Safety – Soy Sauce (GB 2717-2003). Similar GB Standards exist for fermented vinegars (GB2719-2018) and fermented alcoholic beverages (GB 2758-2012). Recently China's State Administration for Market Regulation (SAMR) issued a series of warnings and directives relating to the consumption of FFs, counterfeit foods and beverages, with particular emphasis on the safety of the former, after nine members of a single family in Heilongjiang province died from a single food poisoning event in 2020, determined to be caused by high concentrations of the respiratory toxin bongkrekic acid produced by *Pseudomonas cocovenenans* in homemade suantangzi (fermented corn noodles). Such an incident highlights the need for Standards and regulations relating to FFs, especially in Asian nations where FFs have traditionally constituted an essential part of the staple diet.

### India

In India, the Food Safety and Standards Act (No. 34 of 2006; amended in No. 13 of 2008) established the Food Safety and Standards Authority of India (FSSAI), which is responsible for surveillance of foods and implementation of the act, and laid down science-based standards for manufacture, storage, distribution, export and import of foods, among others (65) (Figure 1, Supplementary Table 1). Regulation of fermented milks is addressed in the Food Safety and Standards (Food Products Standards and Food Additives) Regulations, 2011. The regulation describes the acceptable compositional (primarily nutritional) standards for a variety of milk and dairy products including curd, which is an Indian fermented milk similar to yogurt. Yogurt is described explicitly as a fermented milk produced by lactic acid fermentation by *L. delbrueckii* subsp. *bulgaricus* and *S. thermophilus*, but allows for addition of *Bifidobacterium bifidum, Lactobacillus acidophilus* or “other harmless lactic acid bacteria” if suitable declarations are made on the label, with microorganisms in the final product needing to be “viable and abundant” (Figures 1, 2). The regulation further describes various additives allowed for yogurt. Compositional standards for various types of cheeses are also described in the document. Another regulation that is relevant for FFs is the Food Safety and Standards (Health Supplements, Nutraceuticals, Food for Special Dietary Use, Food for Special Medical Purpose, Functional Food and Novel Food) Regulations, 2016, which became active from January 1, 2018 (Figure 1, Supplementary Table 1). While FFs are not explicitly mentioned in the regulation, “food with added probiotic ingredients” and “novel Foods” are two relevant categories for FFs with health claims. Similar to the EU, India mandates that “probiotic” labeling has to be associated with a health claim and must be backed up by substantial scientific evidence that is evaluated by the FSSAI; the regulation also defines the range of acceptable health claims. Foods with probiotic ingredients must have a minimum viable number of microbes at > 10^8^ CFU/g. The regulation also outlines specific labeling requirements for such foods. The list of approved probiotic organisms is provided in Schedule VII of the regulation and is periodically updated. Novel foods are broadly defined as those without any history of human consumption or those produced through innovative technologies to, for example, alter nutritional value or reduce levels of undesirable substances. These cannot be imported without FSSAI approval. Health claims made for novel foods are also subject to the regulation.

### Republic of Korea (South Korea)

The Republic of Korea has several laws and ministries that are involved in the regulation of foodstuffs. With respect to FFs, the most important legislations include the Food Sanitation Act, the Food Code, Functional Health Foods Act (FHFA), Health Functional Food Code, and the Food Labeling and Advertisement Act (Figure 1, Supplementary Table 1). While multiple major Ministries including the Ministry of Agriculture, Food and Rural Affairs (MAFRA) and the Prime Minister's Office (PMO) are involved in regulation of foods, the Ministry of Food & Drug Safety (MFDS) is the Government agency that is primarily responsible for the establishment and enforcing of food regulations, including setting standards and specification for livestock products, functional foods, food additives, food packaging and equipment.

Standards for a diverse range of FFs, including traditional Korean FFs, are controlled under the Food Sanitation Act (FSA) and covered in the Food Code (pursuant to Article 7(1) of the FSA) (66) (Act No.10787, 07. Jun, 2011, Partial Amendment) (Figure 1, Supplementary Table 1). These Standards include the specifications for foods across their lifecycle including, but not limited to, manufacturing, processing, cooking, packaging, storage, labeling, and distribution. The Code also specifies chemical and microbiological contaminant levels as well as testing methods (MFDS Notification No.2021-54, 2021.6.29.). Kimchi, a globally popular traditional Korean fermented food, is defined in the Korean Food Code as “..made by using vegetables, such as Korean cabbage, etc., as main ingredients, and processing them with/without fermentation after pickling and seasoning mixing process; and kimchi seasoning used for manufacturing kimchi” (Food Code 5.14.1). Ingredients for kimchi, heavy metal and microbiological specifications, and analytical methods are further defined in the document; aflatoxin levels are restricted to <10 parts-per-billion (66). Section 5.9.6 of the Food Code describes different types of fermented beverages, where such beverages are defined as products of fermentation of milk or ingredients of plant origin by lactic acid bacteria or yeasts, with possible processing (pasteurization). Excluding pasteurized products, a standard of at least 10^6^ CFU/ml of lactic acid bacteria/yeasts has been set for the fermented beverages; additional descriptions include standards for counts of undesirable microbes and analytical methods. No specific mention of kombucha is made in the Food Code. Fermented milks, described in Section 5.19.4 of the Food Code, are defined as “products made by fermenting raw milk or milk products with LAB or yeasts; or by adding food or food additives to such fermented milk products.” Further clarification with respect to different types of fermented milks is provided, including for fermented milk (>3% non-fat milk solids), thick fermented milk (>8% non-fat milk solids), fermented cream (>3% non-fat milk solids and >8% milk fat), thick fermented cream (>^*^% non-fat milk solids and >8% milk fat), and fermented buttermilk with >8% non-fat milk solids. A minimum count of 10^7^ CFU/ml of LAB or yeasts is specified for all fermented milk types, along with other microbiological standards, ingredients and analytical methods. Section 5.20.2 of the Food Code, attributed to salted and fermented seafood products, provides the following definition “..products made by adding salt to fishes, crustaceans, mollusks or echinoderms etc., and fermenting and aging them; or by adding food or food additives to the filtrate separated from such fermented and aged foods and processing them. It includes *jeotkal* (salted and fermented seafood), *seasoned jeotkal* (salted-fermented-and-seasoned seafood), fish sauce, and seasoned fish sauce”. *Jeotkal*, seasoned *jeotkal*, fish sauce and seasoned fish sauce are separately defined as well. The Food Code additionally describes ingredients, analytical methods, and microbiological and organoleptic specifications for salted and fermented seafood products. Definitions for fermented soybean products such as *meju* (fermented soybean lump), *doenjang* (fermented soybean paste), *gochujang* (fermented hot pepper soy paste), *chunjang* (fermented black soybean paste), *chungukjang* (fast-fermented soybean paste) and mixed fermented pastes are provided in Section 5.12 of the Food Code. For each food type, ingredients, fermentative process, fermenting microbes (*Bacillus* sp. and *Aspergillus* sp.), processing, and organoleptic standards are specified. Additionally, analytical methods and tests for microbiological standards, tar colors, and total nitrogen content, among others, are described.

In South Korea, functional health foods are defined as “foods manufactured (including processing) with functional raw materials or ingredients beneficial to the human body” in the Functional Health Foods Act (FHFA) (Act No. 12669, May 21, 2014) (Figure 1, Supplementary Table 1). The Health Functional Food Code (HFFC) contains standards and specifications for manufacturing, processing, production, import, distribution, and storage, among others, for functional foods pursuant to Article 14 of the FHFA as well as specifications for functional ingredients themselves pursuant to Article 15 of the FHFA (MFDS Notification No. 2020-92, September 23, 2020). Functional ingredients are only added to the code upon 6 years of recognition through the Regulation on Approval of Functional Ingredients for Health Functional Food along with 50 item manufacturing reports. Probiotics are included as functional ingredients in the HFFC (Section 3.2.51), where 20 probiotic species (not strains) are listed. These may be added to foods, including FFs, to market foods with health claims. Probiotics allowed for functional use include multiple *Lactobacillus* and *Bifidobacterium* species along with *Lactococcus lactis, S. thermophilus* and *Enterococcus faecalis*. Importantly, the only health claim approved for these probiotic functional ingredients is “..may help to increase the number of beneficial bacteria and control harmful bacteria in the gut help to maintain healthy bowel function, maintain gut health” (MFDS Notice 2020-63), with a recommended intake of 10^8^-10^10^ CFU/g; probiotic species and amount in the food must be clearly mentioned in the label. A minimum of 10^8^ CFU/g of live bacteria is recommended for functional foods using probiotics as functional ingredients, with manufacturing/processing specifications to be held identical to “fermented milks” in the Food Code.

## Fermented Foods Regulatory Frameworks in Australia and New Zealand

In Australia and New Zealand, the Australia New Zealand Food Standards Code (ANZFSC) is the overarching legislation that regulates the safety, production, transport, storage and processing of all foods, including FFs (Figure 1, Supplementary Table 1). This is developed and administered by Food Standards Australia New Zealand (FSANZ), an Australian statutory agency within the Australian Government Health portfolio. However, enforcement of the ANZFSC is the responsibility of State and Territory authorities. The Standards in the ANZFSC are legislative instruments (67), with provisions of the code applicable in New Zealand incorporated in the Food Act 2014 (68). Part 2.5 of the ANZFSC describes food standards for dairy products including fermented milk products (Standard 2.5.3) and cheese (Standard 2.5.4). Yogurt is described as a product of fermentation of dairy by lactic acid bacteria in Standard 2.5.3 (version: F2015L00413) (Figures 1, 2), whereas lactic acid bacteria are not specified while defining fermented milk. The Standard additionally describes the pH, microbial count and compositional features for fermented milks and yogurt (Figures 1, 2). Importantly, these standards also apply to products where fermented milk/yogurt is an additional component. Plant sterol addition to fermented milk products is restricted between 0.8 and 1 g/200 g of packed product. Cheese is described in Standard 2.5.4 (version: F2015L00414) as the ripened or unripened solid or semi-solid milk product, whether coated or not, that can be obtained by rennet mediated methods or processing techniques that produce materials with similar characteristics as the former. The addition of tall oil phytosterol esters is restricted to 70–90 g/kg of cheese. Standard 2.2.1 (version: F2016C00173) of the ANZFSC provides guidance regarding labeling requirements for processed, manufactured and unpackaged fermented comminuted meats. Guidelines for acceptable microbiological levels in foods, including fermented foods, can be found in Standard 1.6.1 (version: F2021C00899) of the ANZFSC with elaborations in Schedule 27 (version: F2021C00605). ABT cultures, where the primary microbes are *L. acidophilus, Bifidobacterium* and *S. thermophilus*, are permitted for yogurt compositions in Australia (59). No specific regulation for any other FF is provided in the Code.

Part 1.5 of the ANZFSC describes standards for foods that require pre-market clearance and includes novel foods (Standard 1.5.1). Novel foods are described in Standard 1.5.1 (version: F2017C00324) as non-traditional foods that require an assessment of public health and safety considerations having regard to source, patterns and levels of consumption, composition of food, preparatory process and potential for adverse effects, among others. Non-traditional foods are further defined as either (a) a food that does not have a history of human consumption in Australia or New Zealand; (b) a substance derived from a food, where that substance does not have a history of human consumption in Australia or New Zealand other than as a component of that food; or (c) any other substance, where that substance, or the source from which it is derived, does not have a history of human consumption as a food in Australia or New Zealand. Importantly, both new probiotic microorganisms and foods produced from new sources are listed as novel food categories, where non-traditional and functional fermented foods are eligible under this regulation through the latter. Currently permitted novel foods are listed in Schedule 25 (version: F2021C00564) of the ANZFSC. Although docosahexaenoic acid-rich dried marine algae (*Schizochytrium* sp.) is listed as a novel food, to date, no live probiotics or FFs have been listed as novel foods in Australia-New Zealand. Similar to Europe, improved lactose digestion for live yogurt cultures in lactose intolerant individuals (in fermented milk or yogurt, containing at least 10^8^ CFU/g of *L. delbrueckii* subsp. *bulgaricus* and *S.s thermophilus*) is the only accepted health claim for live microbes (Schedule 4; version: F2017C00711) in Australia-New Zealand.

As for several countries mentioned above, there can be confusion with respect to some FF regulations in Australia and New Zealand. Kombucha regulations in Australia can be considered an apt example of the ramifications of a lack of harmony with respect to legislation for FFs. Indeed, in a recent stakeholder meeting between Government officials and industry members involved in kombucha production in Australia, a separate Fermented Beverage License (FBL) was proposed for manufacturers of kombucha (13). In brief, kombucha fermentation has two stages of fermentation with alcohol being produced in both and secondary fermentation that occurs after bottling in some cases can lead to higher alcohol percentages in the final drink. A national survey for compliance showed that ~77% of kombucha produced in Victoria, Queensland, New South Wales, Tasmania, and South Australia had <1.15% ABV. However, although liquor is defined as being a beverage that contains >1.15% ABV in majority of jurisdictions, Queensland, Tasmania and Victoria consider beverages with ABV > 0.5% as alcoholic. This meant that despite not being a “liquor,” kombucha produced by compliant producers was designated as such in most states and was subject to additional excise and/or liquor duty/licensing. The absence of laws specific for kombucha and disharmony in Liquor laws between states therefore negatively affected businesses; consequently, a proposal to move non-alcoholic beverages to <1.15% ABV nationally has been made (13). The FBL has been proposed to be applicable for microbiologically active beverages such water kefir, jun, kvass, kefir, switchel, apple cider vinegar, fruit vinegars containing yeast, and kombucha. Additional provisions for the FBL includes (i) exemption from the need for Excise or Liquor licensing, (ii) exemption from labeling alcoholic fermentation of beverages, traditional or otherwise, with <1.15% ABV as alcoholic, (iii) requirement for a HACCP safety plan and (iv) registration as a high-risk food business, among others (13). US-based trade association, KBI, has also recently engaged the New Zealand Government for establishing ethanol testing methods for kombucha and held discussions to collaborate with Australia through the ANZFSC. In Australia, the association aims to achieve harmonization of kombucha laws across all States and Territories, allowance of alcohol levels of up to 1.15% ABV for kombucha, establishment of analytical standards for ethanol in kombucha through the ANZFSC, and galvanization of a Australia/New Zealand KBI Committee.

## Fermented Foods Regulatory Frameworks in South America

### Argentina

In Argentina, the Argentine Food Code (Codigo Alimentario Argentino in Spanish; CAA), which was created as an Annex to National Law 18284/69 and put into force by Regulatory Decree 2126/71 in 1971 (69, 70), regulates both locally produced and imported foods, including FFs and has been a reference point for several Latin American countries since the 1970s (70) (Figure 1, Supplementary Table 1). The CAA iteratively incorporates harmonized standards from the Southern Cone Common Market (MERCOSUR; *Mercado Comun Del Sur* in Spanish, includes Argentina, Brazil, Paraguay, Uruguay and Venezuela) framework in the form of resolutions; these Standards are in turn influenced from: (i) the Codex Alimentarius, (ii) the EFSA, (iii) Council of Europe, (iv) German Federal Institute for Risk Assessment (BfR), and, (v) the US FDA (70, 71). The CAA is enforced by three entities: the National Service of Agricultural Food Safety and Quality (SENASA) and the National Wine Institute (INV) within the Ministry of Agriculture, Livestock and Fisheries and the National Administration of Drugs, Foods, and Medical Technology (ANMAT) within the Ministry of Health (70, 71). The CAA is constantly updated by the National Food Commission (CONAL), which was created by Decree 815/99 in 1999 and includes representatives from both ministries.

Regulations for fermented dairy products including labeling, sensory, physico-chemical and microbiological requirements, are described in Chapter VIII (Dairy Foods) of the CAA (69). Articles 576 and 578 of the Chapter outline the definitions and regulatory requirements for a variety of fermented dairy products such as yogurt, acidophilus milk, kumis, kefir and curd/coalhada (Figure 1) (69). Being influenced by the Codex Alimentarius, specifications for fermented dairy products in the CAA are highly similar to the Codex Alimentarius standards for fermented milk products (CODEX STAN 243-2003). Indeed, identical microbes and microbial loads are specified for fermented milks in the harmonized regulations, viz. a minimum of 10^7^ CFU/g of lactic acid bacteria in kefir, kumis, acidophilus milk, and yogurt (35) (Figures 1, 2). Regulatory requirements for cheeses, including labeling, classification, microbiological and nutrient content are described in Articles 605, 610-642 and includes multiple annexes and additional MERCOSUR resolutions. Importantly, the CAA is particularly detailed in relation to regulatory specifications for each type of fermented milk products and cheeses compared to other food Standards, with each food treated separately. In 2020, the CONAL has added specific regulations in relation to kombucha to the CAA. Article 1084 bis of Chapter XIII (Fermented Beverages) of the CAA describes kombucha as “..a carbonated non-alcoholic drink, obtained through aerobic respiration and anaerobic fermentation of a must, composed of an infusion of *Camellia sinensis* and sugars, to which is added a consortium of microbiologically active symbiotic bacteria and yeasts, resulting in an acidic and sweet drink” (69). It further describes specifications for yeasts and bacteria involved in fermentation as well as alcoholic content, among others. Apart from those described above, no other FFs or beverages are currently incorporated in the CAA. No legal framework exists in Argentina for foods with probiotics, i.e., functional foods, which have been classified as dietary foods (CAA Chapter XVII) and have not yet been harmonized by MERCOSUR (70). Additionally, neither CAA nor MERCOSUR define novel foods, with novel ingredients and food products (recognized in practice as per Guidance document GMC 26/03) being added to Chapter XX of the CAA (Miscellaneous) (70, 71).

### Brazil

As mentioned above, Brazil is a member of the Southern Cone Common Market (MERCOSUL in Portuguese) and consequently follows the Codex Alimentarius recommendations for regulation of foodstuffs. The regulatory framework for foodstuffs in Brazil is complex. Food regulations issued at the federal level are contained in various kinds of legal documents with different Government agencies and ministries sharing jurisdiction to ensure food safety, registrations and agricultural import regulation (72, 73). Two government institutions – the Ministry of Agriculture, Livestock and Supply (MAPA) and the Ministry of Health through its regulatory body, Agência Nacional de Vigilância Sanitária (ANVISA), are the primary regulators of agricultural products and foodstuffs including FFs, probiotics, as well as novel foods and foods with health claims (72). While ANVISA is involved in the enforcement of regulations pertaining to processed foods, MAPA oversees and enforces a large number of regulations including import and export of agricultural commodities, and good manufacturing practices, among others. A variety of legislations have been passed by various entities that impact regulation of FFs and beverages as well as functional foods; some of these are discussed here.

Microbiological standards for dairy products, including fermented milks and yogurts, are protected through Resolution RDC n° 331/2019 (amended by RDC n° 459/2020), which refers to the Codex Alimentarius Standards as well as Standard Methods for the Examination of Dairy Products (APHA) for recommendations on sample collection and analysis (74). Resolution RDC n° 359, 2003 was passed by ANVISA to establish portion sizes for packaged foodstuffs including fermented milks and yogurt, thereby providing complementarity with previous resolutions on labeling and manufacture (75). Resolution CNS n° 4, 1988 describes intentional additives allowed in a variety of foodstuffs including fermented milks and yogurt; allowed additives include supplements for improved aroma, natural and artificial coloring, thickeners such as alginic acid, bean gum, agar, and guar gum, among others. Ordinance n° 13/11 by ANVISA also allows Konjak gum to be added as a stabilizer or thickener to yogurts. Resolution RDC n° 51/2010 specifies the use of simulants intended for assays on yogurt and fermented milk packaging (76), while Ordinance 4/1978 from the MAPA through the Department of Animal Origin Products Inspection Service (DIPOA) establishes the manufacturing requirements for dairy products such as recommendations for production facilities, location hygiene, transportation and audit, among others. Brazil also has extensive regulations for cheeses, particularly for its diversity of artisanal cheeses; these regulations have been discussed in greater detail elsewhere (73). Important among these is the ARTE seal (short for *artisanal* in Portuguese) for cheeses, which allows the interstate transport and sale of the products without restriction, provided they have been inspected by Federal or State Agencies (77).

MAPA has recently published the kombucha Identity and Quality Standard (PIQ), which is regulated by Normative Instruction (IN) n° 41 of September 17, 2019. This standard is the first Standard of Identity adopted for kombucha in the world and was developed in conjunction with the Associação Brasileira de Kombucha (ABKOM); KBI was also involved (78). The Brazilian Standard of Identity provides definitions for kombucha with additional description of composition (essential and optional ingredients), classifications, and recommendations for labeling, prohibitions, and analytical parameters. Analytical parameters described in IN n° 41/2019 include a pH range of 2.5–4.2, volatile acidity of 30–130 mEq/L, alcoholic grading of kombucha without (up to 0.5%) and with alcohol (0.6–0.8%) as well as pressure in the kombucha preparation (atm 20°C) added with CO_2_ (1.1–3.9 atm). Labeling recommendations include clearly stating the pasteurization status of the drink, alcohol content, and prohibits use of expressions such as “craft, familiar, homemade, probiotic drink, elixir, elixir of life, premium, energizing, invigorating, live drink,” among others. Overall, unauthorized attribution of superlative characteristics, i.e., functional or health claims are not allowed in labels (78). Additionally, IN n° 41/2019 does not allow the intentional addition of probiotic microbes to kombucha after pasteurization.

Novel and functional foods regulations in Brazil have seen major developments in recent years and are relevant for functional FFs. ANVISA Resolution RDC n° 240/2018 (amends previous RDC n° 27/2010) requires that “novel foods and novel ingredients,” “Bioactive substances and probiotic with functional claims and or health properties claims” and “Food with functional claims and/or health properties claims,” among others, must obtain pre-market clearance (79). In Brazil, novel foods and ingredients are defined as having no history of consumption in the country or being consumed in a modified form or at much lower levels than before (80). Guidelines for the registration of novel foods and functional foods with health claims are established through Resolution n° 16/99 (amended by RDC n° 243/2018) and 19/99 (81), respectively (Figure 1, Supplementary Table 1). Additionally, guidelines for risk assessment and food safety for novel foods is provided by Resolution RDC n° 17/99 (associated guidance document: Guide n° 23, version 1, of 7/23/2019), while guidance on safety, analysis and proof of health claims made in labeling for functional foods is available through Resolution n° 18/99 and Ministerial Order 398/99 (82) (Figure 1, Supplementary Table 1). These foods also need to follow standards (for example, allowed levels of microbiological or chemical contaminants) applicable to normal foods such as Resolution RDC n° 331/2019 and Resolution RDC n° 51/2010, among others. In Brazil, probiotics used in foods with health claims, including functional FFs, need to prove lineage specific health benefits with specified amounts of added/present microbes in target populations (viz., elders, infants, etc.) along with strain viability and stability during gastrointestinal transit (83–85). Resolution RDC n° 241/2018 (guidance document: Guide n° 21/2019) provides guidelines for evaluation of safety and health claims for probiotic microbes for use in food (72). The complex regulatory ecosystem of Brazil has led to the rejection of 100 out of 211 applications for health claims in functional foods by ANVISA, with 66 deferred. To counter this, the Gerência-Geral de Alimentos (GGALI) has been entrusted with defining a method to generate a positive list of probiotics. Once such a list is generated, probiotics on the list will be available for general use including incorporation in foods, including FFs, thereby reducing regulatory challenges faced by manufacturers of functional foodstuffs. Such regulatory reform is aimed at facilitating agile-decision making, minimizing regulatory unpredictability for manufacturers, building customer confidence as well as encouraging public-interest driven innovation and its subsequent translation. Ultimately, it is yet another example of divergent approaches taken by nations in regulating the emergent FFs sector.

## Fermented Foods Regulatory Frameworks in Africa

### South Africa

In South Africa, three Government Ministries are responsible for food legislation: The Department of Agriculture, Forestry and Fisheries (DAFF), the National Department of Health and the Department of Trade and Industry. There is no single Food Law in South Africa, with various laws available for different categories of foodstuffs, viz., Agricultural Products Standards Act (Act No. 119 of 1990), Meat Safety Act (Act No. 40 of 2000), and the Liquor Products Act (Act 60 of 1989); several Acts have a plethora of overlapping legislations (Figure 1, Supplementary Table 1). Consequently, FFs are often regulated through legislations in separate Acts with some overlap. For instance, South Africa has two relevant regulations related to enforcement of yogurt standards. The amended Foods, Cosmetics and Disinfectants Act, 1972 (Act No. 54 of 1972: R.429) forbids the use of the word “probiotic” on labels for yogurt products (86). The legislation allows a functional claim linked to microbial composition in yogurts to be made only when the yogurt culture is comprised of *L. delbrueckii* subsp. *bulgaricus* and *S. thermophilus*. The wording for the functional claim is also specified: “yogurt cultures improve lactose digestion in individuals who have difficulty in digesting lactose (milk sugar)”; this statement can only be appended to yogurt products when the yogurt cultures in yogurt are at > 10^8^ CFU/g (86). The second law, Agricultural Products Standards Act (R.1510; Act No. 119 of 1990), from the DAFF, contradicts the former regulation in certain manners (Figure 2) (87). An amendment of South Africa's Agricultural Product Standards Act, 1990, R.1510 defines yogurt culture as a culture consisting of *L. delbrueckii* ssp. *bulgaricus* and *S. thermophilus* or other similar cultures intended for the manufacture of yogurt (Figure 1). The legislation requires at least 10^7^ CFU/g of yogurt culture in final yogurt/drinking yogurt products. The Standard also allows addition of specific microbes besides yogurt cultures to yogurts, where each supplemented microbe must be present at a viability of 10^7^ CFU/ml with appropriate mention on the label. Additional microbes added beyond the yogurt culture are to be present at a minimum viability of 10^6^ CFU/g. Taxonomic nomenclatures for supplemented microbes are allowed on labels, but “probiotic” labels are not. Overall, although both R.429 and R.1510 are restrictive on “probiotic” claims on the label, they have critical differences regarding allowance of microbial supplementation and minimum yogurt culture viability. An immediate harmonization of these laws are therefore required to provide a uniform regulatory framework vis-à-vis yogurt in South Africa. R.1510 also provides definitions and standards for kefir and maas (also called “amazi” or “amasi”), two cultured milk products. In case of kefir, starter cultures made of bacteria such as *L. kefiri, Leuconostoc* sp., *Lactococcus* sp., and *Acetobacter* sp., as well as lactose fermenting and non-lactose fermenting yeasts are specified, along with a minimum of 10^7^ CFU/g of viable lactic acid bacteria (at least 10^4^ CFU/g of yeasts in case of kefir). R.1510 also specifies standards for various cheeses, along with packaging requirements for dairy products.

## Future Outlook

The current regulatory frameworks for FFs, particularly outside fermented dairy products, are, in general, not mature enough to adequately regulate the significant diversity of FFs that are increasingly available in the market. Indeed, for several FFs, relevant regulations are simply absent. This is particularly true for artisanal FFs and functional FFs (both can also be novel foods). Furthermore, the legislative efforts that have been made have been largely reactive, rather than being proactive, in nature. It is also clear that there is a lack of coherence with respect to such legislation at international, federal and even regional levels. Examples would include, as discussed above, fragmented regulations for the same fermented product at different levels of the federal structure in Australia, or overlapping legislations and numerous resolutions, as observed for Brazil and South Africa. This can, understandably, cause confusion among manufacturers and, indeed, hinder implementation of the legislation. Despite this, it is clear that consolidation of various standards and specifications into legislations and Standard Codes to a certain degree, as done recently by South Korea and India, can provide better harmonization throughout the federal governmental structure.

In our opinion, each fermented product (or at least those resulting from similar fermentative processes) merit specific regulation, outlining specifications for composition, safety, communication and distribution. Some progress in this regard is visible with new legislations for non-dairy fermented products such as kombucha across various countries and the Codex Alimentarius Regional Standards for diverse FFs, as mentioned above. The latter provides a blueprint for developing harmonized regulations for various FF clusters. This is a pragmatic approach too, since most FFs involving similar fermentative procedures tend to be traditionally consumed in specific geographical regions. Furthermore, since many traditional FFs are now globally produced/exported, Codex Standards developed to this end can in turn be implemented in other countries to bring about global harmonization. Importantly, precedent for such implementation using the Codex Alimentarius Standards as guidelines already exists, particularly for fermented milk products; examples include the Argentine CAA as mentioned above, among others. Besides better harmonization of legislations, simpler procedures for approval from relevant authorities and availability of extensive guidance documents are also important instruments in attracting investment and encouraging innovation and commercialization. Other issues include a visible lack of consideration of the insights gained from the large corpus of microbiome studies on FFs and their microbial composition in corresponding global Food Standards or Codes, including the Codex Alimentarius. More must be done to ensure that knowledge gleaned from studies is incorporated into Standards, public policies and legislations. To this end, governments and organizations should consider the establishment of Expert Committees on FF microbiomes to facilitate the smooth translation of such knowledge into public policy recommendations. Finally, legislation for FFs with significant innovative potential, such as functional FFs, should not be frozen in time and must be updated regularly on the basis of robust, newer insights.

## Conclusion

In this review, we have discussed the rapidly evolving global regulatory frameworks for FFs with special emphasis on functional FFs and novel foods, along with the unique legislative bottlenecks and possible resolutions. While some progress has been made in recent years in the development of regulations for FFs, these have mostly been restricted to certain types of FFs (viz., fermented dairy). To preserve consumer confidence in FFs, urgent regulatory advances, including improved regulatory clarity, consistency and harmonization, need to be made to guide consumers on recommended compositions, intakes and to ensure safe production, storage, transport and distribution, among others. Over the years, a lack of understanding of the microbial and chemical composition of fermented products or the absence of appropriate methods to assess relevant safety metrics may have created impediments in the development of such legislations. With recent advances in high-throughput technologies such as genomics and metabolomics, and the resultant availability of suitable testing methods and data from an increasingly higher number of meta-analyses, we anticipate that evidence-based, targeted and harmonized legislations could be swiftly developed, at least for FFs with significant market shares. Indeed, such legislation would be particularly important for regulation of certain novel foods (including traditional FFs) and FFs with health claims, i.e., functional FFs. Importantly, care must be taken to ensure a continuous translation of available evidence to incrementally improve relevant regulations. Ultimately, addressing the challenges outlined here, would contribute to the ease of doing business, encourage consumer and investor confidence, leading to growth and innovation in this category, which in turn will catalyse overall economic progress.

## Author Contributions

AM: conceptualization, methodology, validation, formal analysis, investigation, data curation, and writing—original draft preparation. AM, BG, EO'C, JK, and PC: writing—review and editing. PC: supervision, project administration, and funding acquisition. All authors have read and agreed to the final version of the manuscript.

## Funding

This work, including the article processing charges, was supported by the Institute for the Advancement of Food and Nutrition Sciences (IAFNS) through an ILSI North America Gut Microbiome Committee grant. IAFNS is a non-profit science organization that pools funding from industry and advances science through the in-kind and financial contributions from private and public sector members. IAFNS had no role in the design or presentation of the content of this paper; opinions are those of the authors.

## Conflict of Interest

PC has received funding from Danone, PepsiCo and PrecisionBiotics Group and is sponsored by PepsiCo, H&H, National Dairy Council (USA) to contribute to conferences/meetings. He is also a co-founder and CTO of SeqBiome. The remaining authors declare that the research was conducted in the absence of any commercial or financial relationships that could be construed as a potential conflict of interest.

## Publisher's Note

All claims expressed in this article are solely those of the authors and do not necessarily represent those of their affiliated organizations, or those of the publisher, the editors and the reviewers. Any product that may be evaluated in this article, or claim that may be made by its manufacturer, is not guaranteed or endorsed by the publisher.
