# Supplementary material for: Global Regulatory Frameworks for Fermented Foods: A Review
Source: Front Nutr. 2022 May 23;9:902642. doi: 10.3389/fnut.2022.902642 (PMC9198641; doi:10.3389/fnut.2022.902642)
Supplement: Supplementary file 1 [file Table_1.DOCX]

**Supplementary Table S1. Key Regulations and Standards for fermented foods in various countries.**

| **Country** | **Regulations, Legislations, and Standards relevant to fermented foods*** | **Competent authorities*** | **Reference** |
| --- | --- | --- | --- |
| European Union | - General Food Law Regulation **(Regulation (EC) No 178/2002; current consolidated version 26 July, 2019)** - Regulation (EU) 2015/2283 (current consolidated version 27/03/2021) on novel foods - Nutrition and Health Claims Regulation (Regulation (EU) No 1924/2006) | The European Parliament and Council of the European Union, the European Food Safety Authority (EFSA) | (European Parliament and Council of the European Union, 2015;Bell et al., 2017) |
| Russian Federation | - Article 13 and 26 in Hygienic requirements for manufacturing and trafficking of biological active additives to food - Article 12 in Requirements as to Facilities for Ferment and Probiotic Microorganisms production - Technical Regulations for Milk and Milk Products | Government of the Russian Federation | (Russian Federal Law, 2010;Gaskins et al., 2017) |
| USA | - 21 CFR 131.200 (Subpart B: “Requirements for specific standardized milk and cream”) - “*Draft Guidance for Industry: Acidified Foods”* (75FR50268) - Homemade Foods or Cottage Foods legislations in various US States - Wyoming Food Freedom Act, 2015 - 21 CFR 101 “Food Labeling” | US Food and Drug Administration, US Department of health and Human Services, US Federal Government, US State Governments | (US Food and Drug Administration, 2009;Nyanzi et al., 2021) |
| Canada | - Food and Drugs Act - Food and Drug Regulations - National Dairy Code - Safe Food for Canadians Regulations | Canadian Government, Federal/Provincial/Territorial Food Safety Committee | (Canada, 1985;2012;2022) |
| Japan | - Food Sanitation Act (Act No. 233, 1947) - Food for Specified Health Uses Guidelines - New Functional Products Regulation | Ministry of Health, Welfare and Labour, Government of Japan | (Japan, 1996;Iwatani and Yamamoto, 2019) |
| India | - Food Safety and Standards Act - Food Safety and Standards (Food Products Standards and Food Additives) Regulations, 2011 - Food Safety and Standards (Health Supplements, Nutraceuticals, Food for Special Dietary Use, Food for Special Medical Purpose, Functional Food and Novel Food) Regulations, 2016 | Food Safety and Standards Authority of India, Government of India | (India, 2006) |
| China | - Food Safety Law - New Food Raw Materials Regulation - Guobiao or National Standards | Government of the People’s Republic of China | (Chung and Wong, 2013;Laulund et al., 2017) |
| South Korea | - Food Sanitation Act - Food Code - Functional Health Foods Act - Health Functional Food Code - Food Labeling and Advertisement Act | Ministry of Food & Drug Safety, Ministry of Agriculture, Food and Rural Affairs, Prime Minister’s Office | (Lee et al., 2012) |
| Australia & New Zealand | - Australia New Zealand Food Standards Code | Food Standards Australia New Zealand, Government of Australia and New Zealand | (Australia and New Zealand, 2016) |
| Argentina | - Codigo Alimentario Argentino (enforced by Regulatory Decree 2126/71 in 1971) | Ministry of Agriculture, Livestock and Fisheries, Ministry of Health, National Food Commission, Government of Argentina | (Argentina, 1969;Magnuson et al., 2013;Kirchsteiger-Meier and Baumgartner, 2014) |
| Brazil | - Resolution RDC n^o^ 331/2019 - Resolution RDC n^o^ 359/2003 - Resolution RDC n^o^ 51/2010 - Normative Instruction n^o^ 41 of September 17, 2019 - Resolution RDC n^o^ 240/2018 - Resolution RDC n^o^ 241/2018 - Resolution n^o^ 16/99 - Resolution n^o^ 17/99 - Resolution n^o^ 18/99 - Resolution n^o^ 19/99 | Ministry of Agriculture, Livestock and Supply,  Ministry of Health, Agência Nacional de Vigilância Sanitária, Government of Brazil | (Brazil, 1999a;d;b;c;2018a;b;c;Colombo et al., 2018;Brazil, 2020;Pineda et al., 2021) |
| South Africa | - Foods, Cosmetics and Disinfectants Act, 1972 - Agricultural Product Standards Act, 1990 | Department of Agriculture, Forestry and Fisheries, the National Department of Health and the Department of Trade and Industry, Government of South Africa | (South Africa, 1972;2015) |
| Singapore | - Sale of Food Act - Sale of Food Act (**Food Regulations**) - Health Supplements Guidelines | Agri-Food & Veterinary Authority of Singapore, Health Sciences Authority of Singapore, Government of Singapore | (Kuan, 1996;Ismail, 2011;Tortajada and Zhang, 2016) |
| Malaysia | - Food Act 1983 ("Act") - Food Regulations 1985 ("Regulations") | Ministry of Health Malaysia, Ministry of Agriculture and Agro-Based Industry Malaysia, Government of Malaysia | (Arora et al., 2013;Nadira et al., 2017) |
| Phillipines | - Consumer Act - Food Safety Act of 2013 (Republic Act No. 10611) - Food and Drug Administration Act of 2009 (Republic Act No. 9711) - Milk Code (Executive Order No. 51, National Code of Marketing of Breast milk Substitutes and Other Related Products) | Food and Drug Administration (FDA), Philippine Department of Health, FDA Center for Food Regulation and Research, Department of Trade and Industry, | (Nor et al., 2016) |

* Not an exhaustive list

**References**

Argentina (1969). Código Alimentario Argentino. *Official Bulletin*.

Arora, M., Sharma, S., and Baldi, A. (2013). Comparative insight of regulatory guidelines for probiotics in USA, India and Malaysia: A critical review. *International Journal of Biotechnology for Wellness Industries* 2**,** 51-64.

Australia and New Zealand (2016). Food Standards Australia New Zealand. *New Zealand Gazette*.

Bell, V., Ferrão, J., and Fernandes, T. (2017). Nutritional Guidelines and Fermented Food Frameworks. *Foods (Basel, Switzerland)* 6**,** 65.

Brazil (1999a). Resolução RES nº 16 de 30/04/1999. *Diário Oficial da União* 82**,** 11.

Brazil (1999b). Resolução RES nº 17 de 30/04/1999. *Diário Oficial da União* 82**,** 11.

Brazil (1999c). Resolução RES nº 18 de 30/04/1999. *Diário Oficial da União* 82**,** 11.

Brazil (1999d). Resolução RES nº 19 de 30/04/1999. *Diário Oficial da União* 82**,** 12.

Brazil (2018a). Resolução da Diretoria Colegiada RDC nº 240 de 26/07/2018. *Diário Oficial da União* 144**,** 96.

Brazil (2018b). Resolução da Diretoria Colegiada RDC nº 241 de 26/07/2018. *Diário Oficial da União* 144**,** 97.

Brazil (2018c). Resolução da Diretoria Colegiada RDC nº 243 de 26/07/2018. *Diário Oficial da União* 144**,** 100.

Brazil (2020). Instrução Normativa (IN nº 76 de 05/11/2020). *Diário Oficial da União* 215**,** 75-77.

Canada (1985). Food and Drugs Act. *Revised Statutes of Canada*.

Canada (2012). Safe Food for Canadians Act. *Statutes of Canada*.

Canada (2022). Food and Drugs Regulations. *Consolidated Regulations of Canada*.

Chung, S.S., and Wong, C.K. (2013). Regulatory and policy control on food safety in China. *J Epidemiol Community Health* 67**,** 476-477.

Colombo, M., Todorov, S.D., Eller, M., and Nero, L.A. (2018). The potential use of probiotic and beneficial bacteria in the Brazilian dairy industry. *J Dairy Res* 85**,** 487-496.

European Parliament and Council of the European Union (2015). Regulation (EU) 2015/2283 of the European Parliament and of the Council of 25 November 2015 on novel foods, amending Regulation (EU) No 1169/2011 of the European Parliament and of the Council and repealing Regulation (EC) No 258/97 of the European Parliament and of the Council and Commission Regulation (EC) No 1852/2001. *Off J Europ Union.* 327**,** 1-22.

Gaskins, A.J., Corvalán, C., and Michels, K.B. (2017). Reply to at Wijayabahu. *American Journal of Clinical Nutrition* 106**,** 707.

India (2006). The Food Safety and Standards Act, 2006. *Gazette of India*.

Ismail, R. (2011). Food and consumer protection: A study on food legislation of selected countries. *Asian Law Institute, Singapore*.

Iwatani, S., and Yamamoto, N. (2019). Functional food products in Japan: A review. *Food Science and Human Wellness* 8**,** 96-101.

Japan (1996). Notice, No. 56 (Published by the Ministry of Health and Welfare on 23 May 1996).

Kirchsteiger-Meier, E., and Baumgartner, T. (2014). Global food legislation: an overview.

Kuan, C.H. (1996). Regulatory status of functional foods in Singapore. *Nutrition reviews* 54**,** S168.

Laulund, S., Wind, A., Derkx, P.M.F., and Zuliani, V. (2017). Regulatory and Safety Requirements for Food Cultures. *Microorganisms* 5**,** 28.

Lee, G.-I., Lee, H.-M., and Lee, C.-H. (2012). Food safety issues in industrialization of traditional Korean foods. *Food Control* 24**,** 1-5.

Magnuson, B., Munro, I., Abbot, P., Baldwin, N., Lopez-Garcia, R., Ly, K., Mcgirr, L., Roberts, A., and Socolovsky, S. (2013). Review of the regulation and safety assessment of food substances in various countries and jurisdictions. *Food additives & contaminants. Part A, Chemistry, analysis, control, exposure & risk assessment* 30**,** 1147-1220.

Nadira, A.F., Rosita, J., Norhaizan, M., and Redzwan, S.M. (2017). Screening of aflatoxin M1 occurrence in selected milk and dairy products in Terengganu, Malaysia. *Food Control* 73**,** 209-214.

Nor, N., Masdek, N., and Sulaiman, N.H. (2016). Functional food business potential analysis in Malaysia, Thailand, Indonesia and the Philippines. *Economic and Technology Management Review***,** 99-110.

Nyanzi, R., Jooste, P.J., and Buys, E.M. (2021). Invited review: Probiotic yogurt quality criteria, regulatory framework, clinical evidence, and analytical aspects. *Journal of Dairy Science* 104**,** 1-19.

Pineda, A.P.A., Campos, G.Z., Pimentel-Filho, N.J., Franco, B.D.G.D.M., and Pinto, U.M. (2021). Brazilian Artisanal Cheeses: Diversity, Microbiological Safety, and Challenges for the Sector. *Frontiers in Microbiology* 12.

Russian Federal Law (2010). Technical Regulations for Milk and Milk Products.

South Africa (1972). Foods, Cosmetics and Disinfectants Act, 1972 (Act No. 54 of 1972): Regulations relating to the labelling and advertising of foods: Amendment. *Government Notice Gazette* 37695**,** 3.

South Africa (2015). Agricultural Product Standards Act, 1990 (Act No. 119 of 1990: Regulation R.260 of 2015): Regulations relating to the classification, packing and marking of dairy products and imitation dairy products intended for sale in the republic of South Africa: Amendment. *Government Notice Gazette* 38615**,** 5.

Tortajada, C., and Zhang, H. (2016). Food policy in Singapore. *Food sciences. Elsevier* 1**,** 1-7.

Us Food and Drug Administration (2009). Food Code. [*http://www.fda.gov/Food/GuidanceRegulation/RetailFoodProtection/FoodCode/UCM2019396.htm*](http://www.fda.gov/Food/GuidanceRegulation/RetailFoodProtection/FoodCode/UCM2019396.htm).
