# Supplementary material for: Global Regulatory Frameworks for Fermented Foods: A Review
Source: Front Nutr. 2022 May 23;9:902642. doi: 10.3389/fnut.2022.902642 (PMC9198641; doi:10.3389/fnut.2022.902642)
Supplement: Supplementary file 2 [file Table_2.DOCX]

**Supplementary Table S2. Codex Alimentarius Standards for Cheeses.**

| **Fermented food (Codex Standard)*** | **Standard type and published by** | **Definitions of fermented foods as per Codex Standards** | **Reference** |
| --- | --- | --- | --- |
| General Standard for Cheese (CODEX STAN 283-1978) | Global Standard prepared by the Codex Committee on Milk and Milk Products (CCMMP) | Cheese is the ripened or unripened soft, semi-hard, hard, or extra-hard product, which may be coated, and in which the whey protein/casein ratio does not exceed that of milk, obtained by: (a) coagulating wholly or partly the protein of milk, skimmed milk, partly skimmed milk, cream, whey cream or buttermilk, or any combination of these materials, through the action of rennet or other suitable coagulating agents, and by partially draining the whey resulting from the coagulation, while respecting the principle that cheese-making results in a concentration of milk protein (in particular, the casein portion), and that consequently, the protein content of the cheese will be distinctly higher than the protein level of the blend of the above milk materials from which the cheese was made; and/or (b) processing techniques involving coagulation of the protein of milk and/or products obtained from milk which give an end-product with similar physical, chemical and organoleptic characteristics as the product defined under (a).  Ripened cheese is cheese which is not ready for consumption shortly after manufacture but which must be held for such time, at such temperature, and under such other conditions as will result in the necessary biochemical and physical changes characterizing the cheese in question.  Mould ripened cheese is a ripened cheese in which the ripening has been accomplished primarily by the development of characteristic mould growth throughout the interior and/or on the surface of the cheese.  Unripened cheese including fresh cheese is cheese which is ready for consumption shortly after manufacture. | (Food and Agriculture Organization of the United Nations, 1978) |
| Standard for Unripened Cheese Including Fresh Cheese (CODEX STAN 221-2001) | Global Standard prepared by the Codex Committee on Milk and Milk Products (CCMMP) | Unripened cheeses including fresh cheeses are products in conformity with the General Standard for Cheese (CXS 283-1978), which are ready for consumption shortly after manufacture. | (Food and Agriculture Organization of the United Nations, 2001) |
| Mozzarella (CODEX STAN 262-2006) | Global Standard prepared by the Codex Committee on Milk and Milk Products (CCMMP) | Mozzarella is an unripened cheese in conformity with the *General Standard for Cheese*  (CODEX STAN 283-1978) and the *Standard for Unripened Cheese Including Fresh Cheese* (CODEX STAN 221-2001). It is a smooth elastic cheese with a long stranded parallel-orientated fibrous protein structure without evidence of curd granules. The cheese is rindless and may be formed into various shapes. Mozzarella with a high moisture content is a soft cheese with overlying layers that may form pockets containing liquid of milky appearance. It may be packed with or without the liquid. The cheese has a near white colour. Mozzarella with a low moisture content is a firm/semi-hard homogeneous cheese without holes and is suitable for shredding. Mozzarella is made by “pasta filata” processing, which consists of heating curd of a suitable pH value kneading and stretching until the curd is smooth and free from lumps. Still warm, the curd is cut and moulded, then firmed by cooling. Other processing techniques, which give end products with the same physical, chemical and organoleptic characteristics are allowed. | (Food and Agriculture Organization of the United Nations, 2006) |
| Cheddar (CODEX STAN 263-1966) | Global Standard prepared by the Codex Committee on Milk and Milk Products (CCMMP) | Cheddar is a ripened hard cheese in conformity with the *General Standard for Cheese* (CODEX STAN 283-1978). The body has a near white or ivory through to light yellow or orange colour and a firm-textured (when pressed by thumb), smooth and waxy texture. Gas holes are absent, but a few openings and splits are acceptable. The cheese is manufactured and sold with or without rind which may be coated. For Cheddar ready for consumption, the ripening procedure to develop flavour and body characteristics is normally from 5 weeks at 7–15 °C depending on the extent of maturity required. Alternative ripening conditions (including the addition of ripening enhancing enzymes) may be used, provided the cheese exhibits similar physical, biochemical and sensory properties as those achieved by the previously stated ripening procedure. Cheddar intended for further processing need not exhibit the same extent of ripening when justified through technical and/or trade needs. | (Food and Agriculture Organization of the United Nations, 1966a) |
| Danbo (CODEX STAN 264-1966) | Global Standard prepared by the Codex Committee on Milk and Milk Products (CCMMP) | Danbo is a ripened firm/semi-hard cheese in conformity with the *General Standard for Cheese* (CODEX STAN 283-1978). The body has a near white or ivory through to light yellow or yellow colour and a firm-textured (when pressed by thumb) texture, suitable for cutting, with few to plentiful, evenly distributed, smooth and round pea sized (or mostly up to 10 mm in diameter) gas holes, but a few openings and splits are acceptable. The shape is flat squared or parallelepiped. The cheese is manufactured and sold with or without hard or slightly moist smear ripened rind, which may be coated. For Danbo ready for consumption, the ripening procedure to develop flavour and body characteristics is normally from 3 weeks at 12–20 °C depending on the extent of maturity required. Alternative ripening conditions (including the addition of ripening enhancing enzymes) may be used, provided the cheese exhibits similar physical, biochemical and sensory properties as those achieved by the previously stated ripening procedure. Danbo intended for further processing need not exhibit the same extent of ripening when justified through technical and/or trade needs. | (Food and Agriculture Organization of the United Nations, 1966b) |
| Edam (CODEX STAN 265-1966) | Global Standard prepared by the Codex Committee on Milk and Milk Products (CCMMP) | Edam is a ripened firm/semi-hard cheese in conformity with the *General Standard for Cheese* (CODEX STAN 283-1978). The body has a near white or ivory through to light yellow or yellow colour and a firm-textured (when pressed by thumb) texture, suitable for cutting, with few more or less round rice to pea sized (or mostly up to 10 mm in diameter) gas holes, distributed in a reasonable regular manner throughout the interior of the cheese, but few openings and splits are acceptable. The shape is spherical, of a flat block or of a loaf. The cheese is manufactured and sold with dry rind, which may be coated. Edam of flat block or loaf shape is also sold without rind. For Edam ready for consumption, the ripening procedure to develop flavour and body characteristics is normally from 3 weeks at 10–18 °C depending on the extent of maturity required. Alternative ripening conditions (including the addition of ripening enhancing enzymes) may be used, provided the cheese exhibits similar physical, biochemical and sensory properties as those achieved by the previously stated ripening procedure. Edam intended for further processing need not exhibit the same degree of ripening when justified through technical and/or trade needs. | (Food and Agriculture Organization of the United Nations, 1966c) |
| Gouda (CODEX STAN 266-1966) | Global Standard prepared by the Codex Committee on Milk and Milk Products (CCMMP) | Gouda is a ripened firm/semi-hard cheese in conformity with the *General Standard for Cheese* (CODEX STAN 283-1978). The body has a near white or ivory through to light yellow or yellow colour and a firm-textured (when pressed by thumb) texture, suitable for cutting, with few to plentiful, more or less round pin’s head to pea sized (or mostly up to 10 mm in diameter) gas holes, distributed in a reasonable regular manner throughout the interior of the cheese, but few openings and splits are acceptable. The shape is of a flattened cylinder with convex sides, a flat block, or a loaf. The cheese is manufactured and sold with a dry rind, which may be coated. Gouda of flat block or loaf shape is also sold without1 rind. For Gouda ready for consumption, the ripening procedure to develop flavour and body characteristics is normally from 3 weeks at 10–17 °C depending on the extent of maturity required. Alternative ripening conditions (including the addition of ripening enhancing enzymes) may be used, provided the cheese exhibits similar physical, biochemical and sensory properties as those achieved by the previously stated ripening procedure. Gouda intended for further processing and Gouda of low weights (< 2.5 kg) need not exhibit the same degree of ripening when justified through technical and/or trade needs. | (Food and Agriculture Organization of the United Nations, 1966d) |
| Havarti (CODEX STAN 267-1966) | Global Standard prepared by the Codex Committee on Milk and Milk Products (CCMMP) | Havarti is a ripened firm/semi-hard cheese in conformity with the *General Standard for Cheese* (CODEX STAN 283-1978). The body has a near white or ivory through to light yellow or yellow colour and a texture suitable for cutting, with plentiful, irregular and coarse large rice seed sized (or mostly 1–2 mm in width and up to 10 mm in length) gas holes. The shape is flat cylindrical, rectangular or of a loaf shape. The cheese is sold with or without1 a slightly greasy smear ripened rind, which may be coated. For Havarti ready for consumption, the ripening procedure to develop flavour and body characteristics is normally, depending on weight, 1–2 weeks at 14–18 °C (for smear development) followed by from 1–3 weeks at 8–12 °C depending on the extent of maturity required. Alternative ripening conditions (including the addition of ripening enhancing enzymes) may be used, provided the cheese exhibits similar physical, biochemical and sensory properties as those achieved by the previously stated ripening procedure. Havarti intended for further processing need not exhibit the same degree of ripening when justified through technical and/or trade needs. | (Food and Agriculture Organization of the United Nations, 1966e) |
| Samso (CODEX STAN 268-1966) | Global Standard prepared by the Codex Committee on Milk and Milk Products (CCMMP) | Samso is a ripened hard cheese in conformity with the *General Standard for Cheese* (CODEX STAN 283-1978). The body has a near white or ivory through to light yellow or yellow colour and a firm-textured (when pressed by thumb) texture suitable for cutting, with few to plentiful, evenly distributed, smooth and round pea to cherry sized (or mostly up to 20 mm in diameter) gas holes, but few openings and splits are acceptable. The shape is a flat cylindrical, flat square or rectangular. The cheese is sold with or without1 a hard, dry rind, which may be coated. For Samso ready for consumption, the ripening procedure to develop flavour and body characteristics is normally from 3 weeks at 8–17 °C depending on the extent of maturity required. Alternative ripening conditions (including the addition of ripening enhancing enzymes) may be used, provided the cheese exhibits similar physical, biochemical and sensory properties as those achieved by the previously stated ripening procedure. Samso intended for further processing need not exhibit the same degree of ripening when justified through technical and/or trade needs. | (Food and Agriculture Organization of the United Nations, 1966f) |
| Emmental (CODEX STAN 269-1967) | Global Standard prepared by the Codex Committee on Milk and Milk Products (CCMMP) | Emmental is a ripened hard cheese in conformity with the *General Standard for Cheese* (CODEX STAN 283-1978). The body has a ivory through to light yellow or yellow colour and an elastic, sliceable but not sticky texture, with regular, scarce to plentiful distributed, mat to brilliant, cherry to walnut sized (or mostly from 1 to 5 cm in diameter) gas holes, but few openings and splits are acceptable. Emmental is typically manufactured as wheels and blocks of weights from 40 kg or more but individual countries may on their territory permit other weights provided that the cheese exhibit similar physical, biochemical and sensory properties. The cheese is manufactured and sold with or without1 a hard, dry rind. The typical flavour is mild, nut-like and sweet, more or less pronounced. For Emmental ready for consumption, the ripening procedure to develop flavour and body characteristics is normally from 2 months at 10–25°C depending on the extent of maturity required. Alternative ripening conditions (including the addition of ripening enhancing enzymes) may be used, provided a minimum period of 6 weeks is observed and provided the cheese exhibits similar physical, biochemical and sensory properties as those achieved by the previously stated ripening procedure. Emmental intended for further processing need not exhibit the same degree of ripening, when justified through technical and/or trade needs. | (Food and Agriculture Organization of the United Nations, 1967) |
| Tilsiter (CODEX STAN 270-1968) | Global Standard prepared by the Codex Committee on Milk and Milk Products (CCMMP) | Tilsiter is a ripened firm/semi-hard cheese in conformity with the *General Standard for Cheese* (CODEX STAN 283-1978). The body has a near white or ivory through to light yellow or yellow colour and a firm-textured (when pressed by thumb) texture suitable for cutting, with irregularly shaped, shiny and evenly distributed gas holes. The cheese is manufactured and sold with or without1 a well-dried smear-developed rind, which may be coated. For Tilsiter ready for consumption, the ripening procedure to develop flavour and body characteristics is normally from 3 weeks at 10–16 °C depending on the extent of maturity required. Alternative ripening conditions (including the addition of ripening enhancing enzymes) may be used, provided the cheese exhibits similar physical, biochemical and sensory properties as those achieved by the previously stated ripening procedure. Tilsiter intended for further processing need not exhibit the same degree of ripening when justified through technical and/or trade needs. | (Food and Agriculture Organization of the United Nations, 1968d) |
| Saint-Paulin (CODEX STAN 271-1968) | Global Standard prepared by the Codex Committee on Milk and Milk Products (CCMMP) | Saint-Paulin is a ripened firm/semi-hard cheese in conformity with the *General Standard for Cheese* (CODEX STAN 283-1978). The body has a near white or ivory through to light yellow or yellow colour and a firm-textured (when pressed by thumb) but flexible texture. Gas holes are generally absent, but few openings and splits are acceptable. The cheese is manufactured and sold with or without1 a dry or slightly moist rind, which is hard, but elastic under thumb pressure, and which may be coated. For Saint-Paulin ready for consumption, the ripening procedure to develop flavour and body characteristics is normally from 1 week at 10–17 °C depending on the extent of maturity required. Alternative ripening conditions (including the addition of ripening enhancing enzymes) may be used, provided the cheese exhibits similar physical, biochemical and sensory properties as those achieved by the previously stated ripening procedure. Saint-Paulin intended for further processing need not exhibit the same degree of ripening when justified through technical and/or trade needs. | (Food and Agriculture Organization of the United Nations, 1968c) |
| Provolone (CODEX STAN 272-1968) | Global Standard prepared by the Codex Committee on Milk and Milk Products (CCMMP) | Provolone is a ripened firm/semi-hard cheese in conformity with the *General Standard for Cheese* (CODEX STAN 283-1978). The body has a near white or ivory through to light yellow or yellow colour and a fibrous texture with long stranded parallel-orientated protein fibres. It is suitable for cutting and, when aged, for grating as well. Gas holes are generally absent, but few openings and splits are acceptable. The shape is mainly cylindrical or pear-shaped, but other shapes are possible. The cheese is manufactured and sold with or without1 a rind, which may be coated. For Provolone ready for consumption, the ripening procedure to develop flavour and body characteristics is normally from 1 month at 10–20 °C depending on the extent of maturity required. Alternative ripening conditions (including the addition of ripening enhancing enzymes) may be used, provided the cheese exhibits similar physical, biochemical and sensory properties as those achieved by the previously stated ripening procedure. Provolone intended for further processing and Provolone of low weights (< 2 kg) need not exhibit the same degree of ripening when justified through technical and/or trade needs. Provolone is made by “pasta filata” processing which consists of heating curd of a suitable pH value, kneading and stretching until the curd is smooth and free from lumps. Still warm, the curd is cut and moulded, then firmed by cooling in chilled water or brine. Other processing techniques, which give end products with the same physical, chemical and organoleptic characteristics are allowed. | (Food and Agriculture Organization of the United Nations, 1968b) |
| Cottage cheese (CODEX STAN 273-1968) | Global Standard prepared by the Codex Committee on Milk and Milk Products (CCMMP) | Cottage Cheese is a soft, rindless1, unripened cheese in conformity with the *General Standard for Cheese* (CODEX STAN 283-1978) and the *Standard for Unripened Cheese Including Fresh Cheese* (CODEX STAN 221-2001). The body has a near white colour and a granular texture consisting of discrete individual soft curd granules of relatively uniform size, from approximately 3–12 mm depending on whether small or large type of curd is desired, and possibly covered with a creamy mixture. | (Food and Agriculture Organization of the United Nations, 1968a) |
| Coulommiers (CODEX STAN 274-1969) | Global Standard prepared by the Codex Committee on Milk and Milk Products (CCMMP) | Coulommiers is a soft, surface ripened, primarily mould ripened cheese in conformity with the *General Standard for Cheese* (CODEX STAN 283-1978) which has a shape of a flat cylinder or sectors thereof. The body has a near white through to light yellow colour and a soft-textured (when pressed by thumb), but not crumbly texture, ripened from the surface to the center of the cheese. Gas holes are generally absent, but few openings and splits are acceptable. A rind is to be developed that is soft and entirely covered with white mould but may have red, brownish or orange coloured spots. Whole cheese may be cut or formed into sectors prior to or after the mould development. For Coulommiers ready for consumption, the ripening procedure to develop flavour and body characteristics is normally from 10 days at 10–16 °C depending on the extent of maturity required. Alternative ripening conditions (including the addition of ripening enhancing enzymes) may be used, provided the cheese exhibits similar physical, biochemical and sensory properties as those achieved by the previously stated ripening procedure. Coulommiers intended for further processing need not exhibit the same extent of ripening when justified through technical and/or trade needs. | (Food and Agriculture Organization of the United Nations, 1969) |
| Cream cheese (CODEX STAN 275-1973) | Global Standard prepared by the Codex Committee on Milk and Milk Products (CCMMP) | *Cream Cheese* is a soft, spreadable, unripened and rindless1 cheese in conformity with the *Standard for Unripened Cheeses Including Fresh Cheeses* (CODEX STAN 221-2001) and the *General Standard for Cheese* (CODEX STAN 283-1978). The cheese has a near white through to light yellow colour. The texture is spreadable and smooth to slightly flaky and without holes, and the cheese spreads and mixes readily with other foods. | (Food and Agriculture Organization of the United Nations, 1973c) |
| Camembert (CODEX STAN 276-1973) | Global Standard prepared by the Codex Committee on Milk and Milk Products (CCMMP) | Camembert is a soft surface ripened, primarily mould ripened cheese in conformity with the *General Standard for Cheese* (CODEX STAN 283-1978), which has a shape of a flat cylinder or sectors thereof. The body has a near white through to light yellow colour and a soft-textured (when pressed by thumb), but not crumbly texture, ripened from the surface to the center of the cheese. Gas holes are generally absent, but few openings and splits are acceptable. A rind is to be developed that is soft and entirely covered with white mould but may have red, brownish or orange coloured spots. Whole cheese may be cut or formed into sectors prior to or after the mould development. For Camembert ready for consumption, the ripening procedure to develop flavour and body characteristics is normally from 10 days at 10–16 °C depending on the extent of maturity required. Alternative ripening conditions (including the addition of ripening enhancing enzymes) may be used, provided the cheese exhibits similar physical, biochemical and sensory properties as those achieved by the previously stated ripening procedure. Camembert intended for further processing need not exhibit the same extent of ripening when justified through technical and/or trade needs. Carre de Camembert is a soft surface ripened cheese with a square shape and which comply with all other criteria and requirements specified for Camembert. | (Food and Agriculture Organization of the United Nations, 1973b) |
| Brie (CODEX STAN 277-1973) | Global Standard prepared by the Codex Committee on Milk and Milk Products (CCMMP) | Brie is a soft surface ripened, primarily white mould ripened cheese in conformity with the *General Standard for Cheese* (CODEX STAN 283-1978), which has a shape of a flat cylinder or sectors thereof. The body has a near white through to light yellow colour and a soft-textured (when thumbs-pressed), but not crumbly texture, ripened from the surface to the center of the cheese. Gas holes are generally absent, but few openings and splits are acceptable. A rind is to be developed that is soft and entirely covered with white mould but may have red, brownish or orange coloured spots. Whole cheese may be cut or formed into sectors prior to or after the mould development. For Brie ready for consumption, the ripening procedure to develop flavour and body characteristics is normally from 10 days at 10–16 °C depending on the extent of maturity required. Alternative ripening conditions (including the addition of ripening enhancing enzymes) may be used, provided the cheese exhibits similar physical, biochemical and sensory properties as those achieved by the previously stated ripening procedure. Brie intended for further processing need not exhibit the same extent of ripening when justified through technical and/or trade needs | (Food and Agriculture Organization of the United Nations, 1973a) |

* CODEX STAN and CXS are equivalent designations

**References**

Food and Agriculture Organization of the United Nations (1966a). Codex Alimentarius. Cheddar (CODEX STAN 263-1966). *WHO*.

Food and Agriculture Organization of the United Nations (1966b). Codex Alimentarius. Danbo (CODEX STAN 264-1966). *WHO*.

Food and Agriculture Organization of the United Nations (1966c). Codex Alimentarius. Edam (CODEX STAN 265-1966). *WHO*.

Food and Agriculture Organization of the United Nations (1966d). Codex Alimentarius. Gouda (CODEX STAN 266-1966). *WHO*.

Food and Agriculture Organization of the United Nations (1966e). Codex Alimentarius. Havarti (CODEX STAN 267-1966). *WHO*.

Food and Agriculture Organization of the United Nations (1966f). Codex Alimentarius. Samso (CODEX STAN 268-1966). *WHO*.

Food and Agriculture Organization of the United Nations (1967). Codex Alimentarius. Emmental (CODEX STAN 269-1967). *WHO*.

Food and Agriculture Organization of the United Nations (1968a). Codex Alimentarius. Cottage cheese (CODEX STAN 273-1968). *WHO*.

Food and Agriculture Organization of the United Nations (1968b). Codex Alimentarius. Provolone (CODEX STAN 272-1968). *WHO*.

Food and Agriculture Organization of the United Nations (1968c). Codex Alimentarius. Saint-Paulin (CODEX STAN 271-1968). *WHO*.

Food and Agriculture Organization of the United Nations (1968d). Codex Alimentarius. Tilsiter (CODEX STAN 270-1968). *WHO*.

Food and Agriculture Organization of the United Nations (1969). Codex Alimentarius. Coulommiers (CODEX STAN 274-1969). *WHO*.

Food and Agriculture Organization of the United Nations (1973a). Codex Alimentarius. Brie (CODEX STAN 277-1973). *WHO*.

Food and Agriculture Organization of the United Nations (1973b). Codex Alimentarius. Camembert (CODEX STAN 276-1973). *WHO*.

Food and Agriculture Organization of the United Nations (1973c). Codex Alimentarius. Cream cheese (CODEX STAN 275-1973). *WHO*.

Food and Agriculture Organization of the United Nations (1978). Codex Alimentarius. General Standard for Cheese (CODEX STAN 283-1978). *WHO*.

Food and Agriculture Organization of the United Nations (2001). Codex Alimentarius. Standard for Unripened Cheese Including Fresh Cheese (CODEX STAN 221-2001). *WHO*.

Food and Agriculture Organization of the United Nations (2006). Codex Alimentarius. Mozzarella (CODEX STAN 262-2006). *WHO*.
